# Cumulative temporal association between assisted reproductive technology and childhood cancer: a systematic review and meta-analysis of observational studies

**DOI:** 10.3389/fonc.2025.1555420

**Published:** 2025-05-20

**Authors:** Yanjie Jiang, Qiangqiang Dai, Shipeng Zhang, Hanyu Wang, Xingyi He, Rui Fu, Junwen Tan, Qinwei Fu, Qinxiu Zhang, Yan Lu

**Affiliations:** ^1^ Neurology Department, Nanjing Hospital of Chinese Medicine Affiliated to Nanjing University of Chinese Medicine, Nanjing, China; ^2^ Hospital of Chengdu University of Traditional Chinese Medicine, Chengdu University of Traditional Chinese Medicine, Chengdu, China; ^3^ Department of Health Research Methods, Evidence, and Impact (HEI), McMaster University, Hamilton, ON, Canada; ^4^ Gynecology Department, World Health Organization Collaborating Centre (WHOCC), Chengdu, China

**Keywords:** assisted reproductive technology, childhood cancer, relative risk, systematic review, meta-analysis, preventive health

## Abstract

**Objective:**

Assisted reproductive technology (ART) has contributed to the birth of over 10 million children worldwide; however, its long-term health impacts, especially the potential risk of childhood cancer, continue to be a subject of debate. This study aims to examine the most current risk associations between ART and childhood cancer.

**Methods:**

We conducted a comprehensive search across PubMed, Embase, Web of Science, and Cochrane Library databases up to August 1, 2024. These studies aimed to explore the association between ART and childhood cancer risk, covering overall cancers, haematological malignancies, neural tumors, other solid tumors, and 11 specific cancers. Pooled analyses of risk estimates and 95% confidence intervals were conducted using random effects models, while cumulative meta-analyses were conducted to provide a time-based summary of the evidence. The study was prospectively registered on PROSPERO (CRD42024547262).

**Results:**

Sixteen large sample observational studies were included. Our findings showed a 21% increased risk of overall cancer in children conceived via ART (relative risk [RR] = 1.21, 95% CI, 1.11–1.33), with elevated risks also noted for haematological malignancies (RR = 1.16, 95% CI, 1.05–1.28), neural tumors (RR = 1.19, 95% CI, 1.07–1.32), and other solid tumors (RR = 1.48, 95% CI, 1.26–1.73). Six specific cancer types also demonstrated higher risks. The direction and magnitude of the effects remained relatively constant over time, while the degree of precision increased as data from newer studies were incorporated. Sensitivity analyses confirmed the robustness of these findings, and no publication bias was found.

**Conclusions:**

Our findings suggest a potential risk association between ART and childhood cancer, raising concerns regarding the future application of ART. These findings are critical in informing infertile couples considering ART about the potential risks involved.

**Clinical trial registration:**

https://www.crd.york.ac.uk/prospero/, identifier CRD42024547262.

## Introduction

Reports indicate that 48 million couples and 186 million individuals worldwide suffer from infertility (1, 2). The social consequences of infertility are significant, as the problem can lead to psychological distress, social stigmatization, economic strain, and even divorce, thereby positioning it as a public health priority (2, 3). This situation has improved with ongoing advancements in medical treatments and technology, enabling millions of infertile couples to conceive through ART (4). Currently, more than 10 million children have been born through ART globally, accounting for 5.1% of all U.S. births (5). Previously, this technology was considered safe; however, the short-term outcomes of children conceived via ART, including fetal birth defects and growth abnormalities, have been well-documented (6–8). However, the medium- and long-term effects of ART on child health remain largely unknown (9). In particular, the association between ART and the risk of childhood cancer has been a subject of ongoing controversy (5). Childhood cancers constitute the second most common cause of childhood mortality in developed countries; however, their etiology remains elusive, and their disease burden is projected to rise significantly, with ART recognized as a possible risk factor (10, 11). Several studies have indicated that certain childhood cancers originate in the early stages of fetal development and that events surrounding conception may play a crucial role in these cancers (9, 12). Given that each stage of ART implementation differs fundamentally from natural conception, it may impact the early developmental stages of the embryo (13, 14). Consequently, several researchers have proposed that ART could be a potential risk factor for childhood cancer (15, 16).

In the 1990s, studies suggested that ART might increase the risk of childhood cancer (17); however, subsequent research revealed no significant risk association between ART and childhood cancer (18), and it may even offer protective benefits (19). The conclusions of these studies lack persuasiveness due to limited sample sizes and variability in research methodologies. Recent large cohort studies investigating the link between ART and childhood cancer have yielded inconsistent and controversial conclusions (20–22). Currently, six published systematic reviews and meta-analyses on this topic have not reached a unanimous conclusion regarding the risk association between ART and childhood cancer, and the opinions are evenly divided into those in favor and those against. In 2005, Raimondi et al. (23) reported that there is no evidence supporting an increased risk of childhood cancer associated with ART. In 2019, a meta-analysis of cohort studies by Gilboa et al. (24) concluded that ART, particularly IVF, is not associated with an overall increased risk of childhood cancer. In 2020, Zhang et al. (25) demonstrated that IVF, intracytoplasmic sperm injection (ICSI), and fertility drugs are not linked to the risk of offspring cancer; however, contrasting results were observed with frozen embryo transfer methods. Conversely, in 2013, Hargreave et al. (15) concluded that fertility treatments might increase the risk of overall childhood cancers, haematological malignancies, neural tumors, and other solid tumors. In 2019, Wang et al. (16) further concluded that ART increases the risk of childhood cancers. Additionally, in 2019, Chiavarini et al. (26) reported a positive association between ART and the risk of childhood cancer. These studies have found some evidence for a link between ART and childhood cancer risk. However, a careful review has revealed methodological flaws, including design limitations and inadequate analytical approaches. For instance, studies with smaller sample sizes may increase the risk of biased results, necessitating revalidation of these findings.

In addition, larger cohort studies conducted over the past four years have introduced new and robust evidence into the field. Notably, two cohort studies (20, 27) encompassing nearly 10 million participants have significantly enriched our knowledge base, enhancing the persuasiveness of our findings. Therefore, the aim of this study was to further assess the impact of ART on childhood cancer and to examine future research trends. This study also aims to provide couples considering ART with the latest information on cancer risk and to assess whether ART should be included as a risk factor for childhood cancer.

## Methods

### Registration of review protocol

This study adhered strictly to the Preferred Reporting Items for Systematic Reviews and Meta-Analyses 2020 (PRISMA) (28) guidelines for a systematic review and meta-analysis of studies examining the relationship between ART and childhood cancer risk. The study protocol was registered and published in the Prospective Registry for International Systematic Reviews (PROSPERO), Registration No. CRD42024547262.

### Search strategy

We conducted searches in the PubMed, Embase, Web of Science, and Cochrane Library databases from inception to August 1, 2024, for relevant publications. The language of the publications was limited to English. The search strategies included terms related to exposure (“Assisted reproductive technology”, “Intracytoplasmic sperm injection”, “*In vitro* fertilization”, “ART”, “ICSI”, “IVF”), specific populations (“infant”, “pediatric”, “childhood”), and outcomes (“Neoplasms”, “Cancer”, “Tumor”) via both MeSH terms and free-text keywords. The reference lists of related articles were manually reviewed to identify any relevant studies missed in the initial search. More detailed information on the search process is available in the Search Strategies for the **Supplementary Material**.

### Eligibility criteria

As recommended, the PECO-S framework was employed to delineate the research questions (29, 30). P-population: children from any country or region; E-exposure: number of children conceived via ART > 4000; C-comparison: unexposed populations, including naturally conceived children, the general population, children not conceived via ART, and naturally conceived children of mothers with low fertility; O-outcome: the outcome of interest was the risk of childhood cancer; S-study design: our focus was on observational study reports, including cohort studies, case-control studies, and cross-sectional studies.

We used the following exclusion criteria (1): duplicates of literature or reports on the same cohort (2); studies with unavailable data (3); studies on the offspring of childhood cancer survivors (4); nonrelevant exposures, such as fertility medications (5); nonrelevant outcomes, such as birth defects and cardiovascular disease in children conceived via ART (6); nonrelevant study designs, such as intervention studies, randomized controlled trials, study protocols, reviews, commentaries, and case reports.

It is noteworthy that, as childhood cancer is a rare disease, several studies have indicated that a sample size of at least 20,000 children is required to observe a doubling of cancer risk within a cohort (31–33). In contrast, most existing large-sample studies (e.g., the Nordic ART registry) have typically included ≥4,000 children conceived via ART. A previous meta-analysis on this topic recommended a minimum sample size of 5,000 children conceived through ART; however, considering both statistical validity and data availability, a threshold of 4,000 was selected (24). This approach encompasses most high-quality studies while mitigating the confounding effects of heterogeneity due to small sample sizes.

### Definition

ART is defined to include all interventions where human oocytes, sperm, or embryos are treated *in vitro* for reproductive purposes (34, 35), including artificial insemination, conventional IVF, ICSI, oocyte and embryo donation, and other forms of treatment; fertility drugs alone are not included in this study.

Childhood cancers are defined with reference to the International Classification of Childhood Cancer (ICCC-3) (36), and classified on the basis of the results of previous studies. The classification encompasses overall cancers, haematological malignancies, neural tumors, other solid tumors, and 11 specific cancers: leukemia [including acute lymphoblastic leukemia (ALL) and acute myelocytic leukemia (AML)], lymphoma, central nervous system tumors (CNS tumors), peripheral nervous cell tumors (including neuroblastoma and other peripheral nervous cell tumors), retinoblastoma, hepatic tumors, renal tumors, bone tumors and extraosseous sarcomas, germ cell tumors, embryonal tumors, and epithelial tumors and melanoma.

### Study selection

Literature screening was conducted in two stages. Initially, two authors (JYJ and ZSP) imported the search results into EndNote X9, removed duplicates, and preliminarily selected studies by reviewing titles and abstracts on the basis of predefined inclusion and exclusion criteria. The second stage involved a detailed review of the full texts to confirm their suitability for the meta-analysis. Any disagreements during the screening process were resolved through discussions with a third author (WHY) until a consensus was reached.

### Data extraction

Two researchers (JYJ and ZSP) independently extracted the data. Disputes were resolved by consulting a third researcher (WHY) or, if necessary, by contacting the study authors for clarification of unclear or missing information. Data on the following study characteristics were extracted: lead author, publication date, study design, geographic location, duration of study, years of follow-up, maternal age at conceiving, type of ART exposure, unexposed population, type of outcome cancer, number of cancers in exposed children/total number of exposed children, number of cancers in unexposed children/total number of unexposed children, RR, 95% confidence intervals, and a list of adjusted covariates. In cases of multiple publications on the same cohort, priority was given to selecting the most comprehensive and up-to-date combined study (with the longest follow-up or analysis covering the most participants).

### Data synthesis and analysis

RR and 95% CI were used as summary indicators of associations. The hazard ratio (HR), odds ratio (OR), and standardized incidence ratio (SIR) are assumed to approximate the same RR metrics because of the lower incidence of childhood cancer. This study analyzed the combined RR via the DerSimonian–Laird random effects model. In addition, according to Wang et al. (16), for treatments that reported data on the risk of different types of cancer within the same category, the RR for the combined cancer category was calculated in pooled analyses using a fixed-effects model in the same study (for details, see Research Data Harmonization). We used the Cochran Q test and the I² statistic to evaluate heterogeneity among studies, with an I² value greater than 50% and a p-value less than 0.05 indicating substantial heterogeneity (37, 38). To assess the extent of heterogeneity, we estimated the 95% prediction interval, which is expected to contain the true effects of 95% of future studies (39, 40). In addition, when the number of studies reporting on a cancer outcome exceeded 10, a cumulative meta-analysis was conducted for that cancer to evaluate the accumulation of evidence over time (41, 42). Additionally, subgroup analyses were conducted using prespecified study-level characteristics, including geographic location, maternal age at conceiving, and various unexposed populations (naturally conceived children, the general population, children not conceived via ART, and children from mothers with low fertility who were naturally conceived), to identify significant factors contributing to the heterogeneity of the primary outcome for this cancer. To ensure the reliability of the naturally conceived population, the general population and children not conceived via ART were combined in the subgroup analyses. In addition, given that childhood cancers encompass various cancer types and ART comprises multiple therapy forms, these factors may also contribute to heterogeneity. Therefore, pooled analyses were performed for individual cancer types, and the associations between different ART types and childhood cancer risk were discussed (the number of studies reporting on some ART exceeded 5). We performed ‘leave-one-out’ sensitivity analyses by removing one study per iteration to examine the impact of individual studies on the overall effect (cancer outcomes with ≥ 5 studies). Funnel plots were visually inspected for publication bias, and Egger regression was used to detect potential publication bias (43); adjustments were made via the trim and fill method if significant bias was identified (44). A two-tailed P value of 0.05 was considered statistically significant.

### Evaluation of study quality

The quality of the studies included in this meta-analysis was assessed via the Newcastle–Ottawa Scale (NOS) (45). The NOS comprises eight items across three dimensions—selection, comparability, and outcome—with each item scoring one point. Specifically, items concerning the control of confounders could receive up to two points. Studies were categorized as low (0–3), medium (4–6), or high (7–9) quality on the basis of their scores. In addition, based on the Grading of Recommendations, Assessment, Development, and Evaluation (GRADE) (46), this study assessed the quality of evidence for each outcome and categorized it as “high”, “moderate”, “low”, or “very low” quality to support the conclusions.

### Software, data, and code availability

Statistical analyses were performed via Stata 16 (Stata Corp, College Station, Texas), and 95% prediction intervals were calculated via the R package “metamisc” in R Studio version (version 4.4.1).

## Results

### Literature search

**Figure 1:**
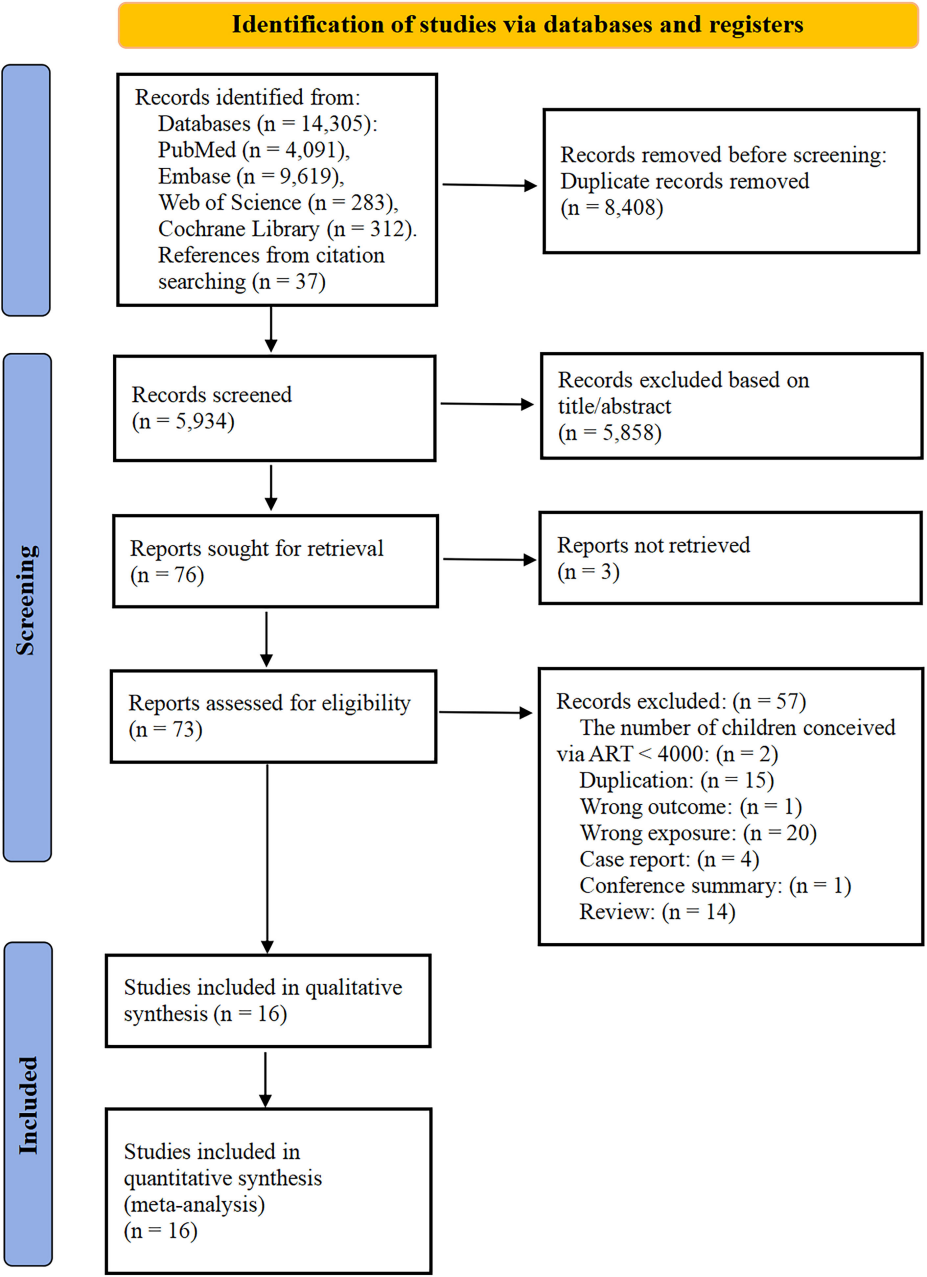
PRISMA flowchart of the systematic search.

**Figure 1** displayed the PRISMA flowchart for this study. A total of 14,342 potentially relevant articles were identified through comprehensive database and citation searches. After removing 8,408 duplicates, a total of 73 documents were deemed eligible for full-text review after screening the titles and abstracts. After further review, 57 records were excluded for the following reasons (**eTable 1).** Ultimately, 16 studies that reported on ART and childhood cancer risk were included in this meta-analysis.

### Characteristics and quality of the included studies

**Table 1** summarized the study designs and demographic characteristics of the included articles, covering 16 large-scale observational studies, which were all classified as cohort studies (10, 11, 18–22, 27, 47–54).

**Table 1:** Selected characteristics of 16 cohort studies included in review.

Lead author, publication date	Geographic region	Study period	Follow-up (years)	Maternal gestational age	Exposure^1^	Unexposed population	Outcome	No. of cancers/ total exposed^2^	No. of cancers/ total unexposed^2^	Covariates adjusted for
F Bruinsma (2000) (54)	Australia	1979-1995	3.75	NA	ART	General population	Overall cancer	6/5249	4.33 (expected)	Age
Ojvind Lidegaard (2005) (53)	Denmark	1995-2002	non-IVF: 4.5 IVF: 4.1	NA	IVF	Naturally conceived children	Kidney cancer, Retinoblastoma, and AML	0/6052	72/442349	None
Bengt Källén (2010) (52)	Sweden	1982–2005	Max: 23	28.73	IVF	General population	All cancers, Hematological cancers, CNS cancers, and Retinoblastomas	53/26692	6405/2417878	Age, maternal age, parity, smoking, and years of unwanted childlessness
Marte Myhre Reigstad (2016) (19)	Norway	1984-2011	ART: 6.9 No ART: 13.7	28	ART	Children not conceived via ART	Overall cancer, Leukemia (ALL, AML, and Other leukemias), Lymphoma (Hodgkin's and Non-Hodgkin's), CNS (Astrocytomas,Embryonal CNS tumors, Other gliomas, and Other CNS tumors), Neuroblastoma, Retinoblastoma, Renal, Hepatic, Soft tissue, and Others	51/25782	4503/1602876	Birth year, birth order, maternal age at delivery, place of birth, gender, birth weight, and gestational age.
Tamar Wainstock (2017) (51)	Israel	1991-2013	10.55	28.16	ART (IVF)	SC	All cancer	48/4324	1450/237863	Maternal age, birthweight, preterm birth, pregnancy-related hypertensive disorders, and pregestational and gestational diabetes mellitus.
Liat Lerner-Geva (2017) (50)	Israel	1997-2011	ART: 10.6 SC: 9.3	28.25	ART	SC	All cancer, Leukemias, myeloproliferative diseases, myelodysplastic diseases, Lymphomas and reticuloendothelial neoplasms, CNS and miscellaneous intracranial and intraspinal neoplasms, Neuroblastoma and other peripheral nervous cell tumors, Retinoblastoma, Renal tumors, Hepatic tumors, Malignant bone tumors, and Soft tissue and other extraosseous sarcomas	21/9042	361/211763	Maternal age, maternal education, ethnicity, plurality, gender, birthweight, and congenital malformations
Carrie L Williams (2013 and 2018) (18, 49)	Britain	1992-2008	6.6/7.86	34.74	ART	General population	All cancers, Leukemia, CNS tumors, Neuroblastoma, Retinoblastoma, Renal tumors, Hepatic tumors, Bone tumors and extraosseous, and Germ-cell tumors	120/118150	124.1 (expected)	Age and gender
Logan G Spector (2019) (48)	United States	2004-2018	4.6	35.87	IVF	Children conceived naturally	Any cancer, Leukemia, ALL, AML, Lymphoma, CNS cancer, Astrocytoma, Ependymoma, Intracranial embryonal tumors, Neuroblastoma, Retinoblastoma, Renal cancer, Hepatic cancer, Soft-tissue sarcoma, and Embryonal tumors	321/146875	2042 /2194854	State of birth, maternal race and ethnicity, maternal educational level (college graduate vs less than college graduate), maternal age, and child’s sex
Marie Hargreave (2019) (47)	Denmark	1996-2015	11.3	30.32	ART (IVF, ICSI, FET)	General population	Any Childhood Cancer, Leukamia, Lymphomas, Central nervous system neoplasms, Sympathetic nervous system tumors, and other cancers	<90/37156	1876/910291	Year of the birth
Barbara Luke (2022) (10)	United States	2004-2018	6.1	29.69	ART	Naturally conceived	All cancers, Leukemia, CNS tumors, Embryonal tumors, and Solid tumors	240/165125	1469/1353440	Conception groups, IVF groups, paternal and maternal ages, maternal race and ethnicity, education, BMI, parity, diabetes, hypertension, plurality, infant sex, and State and year of birth
Nona Sargisian (2022) (27)	Nordic countries: Denmark, Finland, Norway, and Sweden	1984-2018	ART: 9.9 SC: 12.5	29.79	ART (FET)	SC	Any cancer, Leukemia, Lymphomas, Central nervous system tumors, Neuroblastoma and other, peripheral nervous cell tumors, Retinoblastoma, Renal tumors, Hepatic tumors, Bone tumors, Soft tissue sarcomas, Germ cell and gonadal tumors, Epithelial tumors and melanoma, and Other and unspecified tumors	329/171774	16184/7772474	Sex, plurality, year of birth, country of birth, maternal age at birth, and parity
Shiue-Shan Weng (2022) (11)	China	2004-2022	6	30.83	ART	a^3^: Natural conception b^3^: Subfertility and Children not conceived via ART	Overall Cancer, Leukemia, Hepatic tumors, Lymphomas and reticuloendothelial neoplasms, CNS and miscellaneous intracranial and intraspinal neoplasms, Neuroblastoma and other peripheral nervous cell tumors, Retinoblastoma, Renal tumors, Malignant bone tumors, Soft tissue and other extraosseous sarcomas, Germ cell tumors, trophoblastic tumors, and neoplasms of the gonads,and Other malignant epithelial neoplasms and malignant melanomas	47/47152	1833/2260864	Maternal age, paternal age, child’s birth year, child’s sex, parity, socioeconomic status, population density of living areas, and abortion history
Shu Qin Wei (2022)	Canada	2008-2020	6.5	31.24	ART	Children not conceived via ART	Overall Cancer, Leukemia	14/16626	1084/781028	Maternal age, parity, maternal comorbidity, substance use disorders, socioeconomic deprivation, and year of birth
Mandy Spaan (2023) (21)	Netherlands	1983-2019	11.8	33.25	ART (IVF, ICSI, Fresh ET, FET)	Naturally conceived children and children conceived by fertility drugs from subfertile couples.	Overall cancer, Leukemia, Lymphoblastic leukemia, Lymphoma, Breast, Cervix, Testis, Kidney, Brain, and Melanoma	157/51417	201/37832	Birth year
Paula Rios (2024) (20)	France	2010-2023	6.7	30.12	ART (Fresh ET, FET, AI)	Children conceived naturally	Any cancer, Leukemia, ALL, AML, Lymphoma, Malignant CNS tumor, Embryonal tumor, Adrenal gland tumor, Retinoblastoma, Renal tumor, Soft tissue sarcoma, and Epithelial neoplasm and melanoma	292/260236	8964/8266070	Year of birth, sex, multiple birth, maternal age, and deprivation index

^1^Parentheses after ART indicate that this study also explored the relationship between different types of ART and the risk of childhood cancer.

^2^When both overall cancer and other types of cancer were reported in the same study, only data for overall cancer was shown.

AI, artificial insemination; ALL, acute lymphoblastic leukemia; AML, acute myeloid leukemia; ART, Assisted reproductive technology; CNS, central nervous system; ET, embryo transfer; FET, frozen-thawed embryo transfer; ICSI, intracytoplasmic sperm injection; IVF, in vitro fertilization; SC, Spontaneous conception.

^3^The Shu Qin Wei 2022 study included two unexposed populations, a : Natural conception,and b: Subfertility and Children not conceived via ART.

This meta-analysis imposed no stringent publication date restrictions; however, included studies were published between 2000 and 2024. Nine studies originated from Europe (France, the Netherlands, the United Kingdom, Denmark, Finland, Norway, Sweden) (18–21, 27, 47, 49, 52, 53), three from Asia (China, Israel) (11, 50, 51), three from North America (United States, Canada) (10, 22, 48), and one from Oceania (Australia) (54). The average follow-up duration was 7.5 years, and the maternal age at conceiving ranged from 28 to 36 years. In this study, 1,091,652 children were born through ART, of whom 1,789 were diagnosed with cancer. Moreover, in the control group, 28,612,981 children were born, of whom 46,573 were diagnosed with cancer. These groups included children aged 0–18 years. Additionally, except for the studies by Wainstock and Bruinsma et al. (51, 54), the other studies provided detailed reports on the associations between ART and three types of cancers in children: haematological malignancies, neural tumors, and other solid tumors. Eight additional studies (10, 20, 21, 27, 47, 48, 51, 52) investigated the associations between various types of ART and the overall risk of cancer in children. Among the 16 cohort studies, the unexposed people in 8 studies were naturally conceived children (10, 11, 20, 27, 48, 50, 51, 53), 5 studies had general populations (18, 47, 49, 52, 54), 2 studies had children not conceived via ART (19, 22), and 2 studies had mixed populations (11, 21), including low-fertility but naturally reproducing individuals and children not conceived via ART. There were two groups of unexposed populations in the study by Weng et al. (11): one naturally conceiving population and one mixed population. All studies adjusted or matched for potential confounders when estimating the association between ART and childhood cancer risk, with the exception of six studies (18, 21, 47, 49, 53, 54) that differed in the extent of adjustment, as they adjusted only for confounders such as year of birth, age, and sex.

Among the cohort studies included in this study, 15 reported overall cancers, 14 reported haematological malignancies, 12 reported neural tumors, and 13 reported other solid tumors (**eTable 2**).

Additionally, the number of studies reporting on each specific cancer type was as follows: 12 studies reported on leukemia (3 on ALL and 4 on AML), 8 on lymphoma, 12 on CNS tumors, 8 on peripheral nervous cell tumors, 10 on retinoblastoma, 5 on hepatic tumors, 10 on renal tumors, 7 on bone tumors and extraosseous sarcomas, 2 on germ cell tumors, 3 on embryonal tumors, and 4 on epithelial tumors and melanoma (**eTable 2**).

Among the various types of ART, 5 studies have explored the association between IVF and the risk of childhood cancer compared to the general population and unexposed populations conceived naturally.

The 16 studies included in this meta-analysis had NOS scores ranging from 7 to 9. Therefore, the overall quality of this study was rated as high (**eTable 3**).

### ART and overall risk of cancer

**Figure 2:**
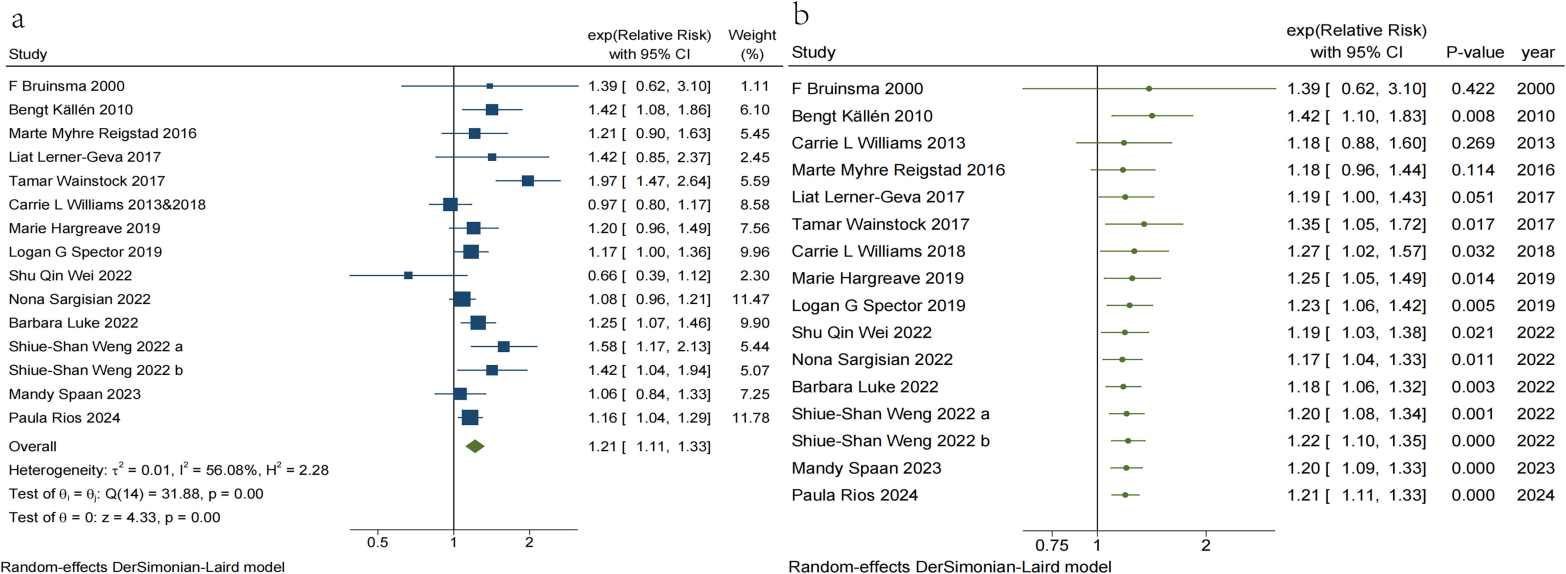
**(a)** Traditional meta-analysis of Risk of Overall cancer in children conceived via ART. **(b)** Cumulative meta-analysis of Risk of Overall cancer in children conceived via ART.

In the present study, we observed a 21% increase in overall cancer risk (RR = 1.21, 95% CI, 1.11–1.33) in children conceived via ART (**Figure 2a**). Heterogeneity was observed among the studies (I² = 56.08%, p = 0.004). We excluded five studies (18, 21, 47, 49, 54) that varied in the degree of adjustment for confounders, resulting in a pooled effect size of (RR = 1.26, 95% CI, 1.14–1.41) (**eFigure Forest Plot 1**). Despite this, heterogeneity among the included studies persisted (I² = 61.87%, p < 0.001). Excluding four studies, two (10, 20) that combined risk estimates for several types of ART and two (48, 52) that employed a single ART risk estimate instead of any ART risk estimate resulted in a pooled effect size of (RR = 1.22, 95% CI, 1.06–1.40) (**eFigure Forest Plot 2**), while significant heterogeneity persisted (I² = 65.77%, p < 0.001). A visual inspection of the funnel plots indicated that the distribution of studies was generally symmetrical, and Egger’s regression test revealed no potential publication bias (t = 1.18, p = 0.258) (**eFigure Publication bias 1–3**). The 95% prediction interval for the pooled analysis ranged from 1.09–1.75, indicating that the true RR for any given study typically lies within this range.

In addition, a cumulative meta-analysis based on year of publication demonstrated (**Figure 2b**) that the first evidence of an increased risk of overall childhood cancer with ART emerged in 2010, with combined RR values ranging from 1.17 to 1.42. However, in 2013, expanded data indicated that the risk association lacked statistical significance. Subsequent research published in 2017 reaffirmed the strong association between ART and overall childhood cancer risk. Although subsequent studies have enhanced the precision of point estimates, there has been no substantial change in the direction or magnitude of the risk association, which has largely stabilized.

### ART and the risk of haematological malignancies, neural tumors and other solid tumors

**Figure 3:**
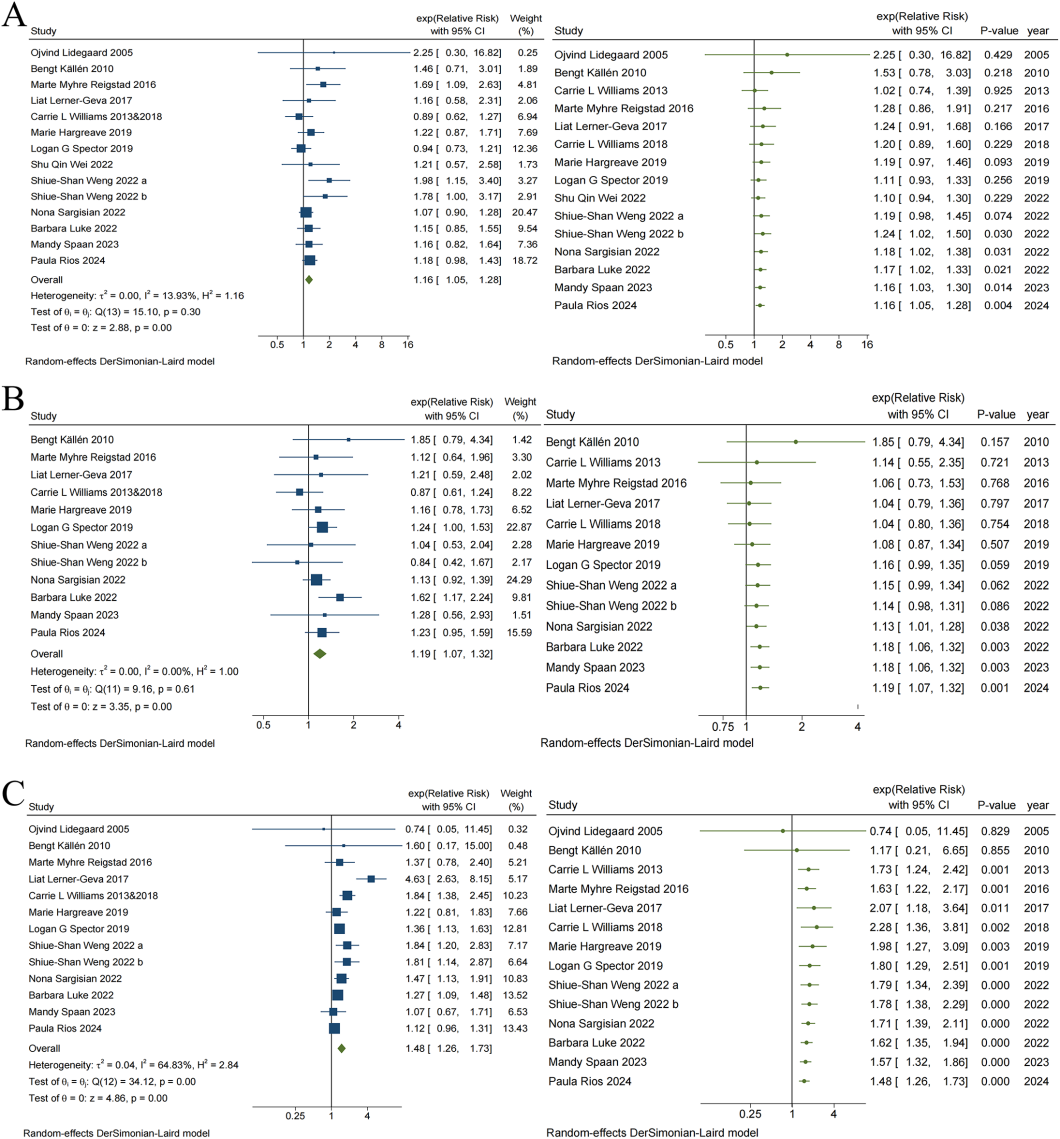
Traditional and Cumulative meta-analysis of Risk of Haematological malignancies **(A)**, Neural tumors **(B)** and Other solid tumors **(C)** in children conceived via ART.

**Figure 3** illustrates the risk associations for haematological malignancies, neural tumors, and other solid tumors associated with ART, respectively. The pooled results indicated that the risk for haematological malignancies (RR = 1.16, 95% CI, 1.05–1.28), neural tumors (RR = 1.19, 95% CI, 1.07–1.32), and other solid tumors (RR = 1.48, 95% CI, 1.26–1.73) significantly increased in children conceived via ART. Heterogeneity was observed only in studies reporting other solid tumors (I² = 64.83%, p < 0.05), and cancer type may significantly influence heterogeneity. The visual funnel plots were symmetrical, and none of the Egger regression tests indicated potential publication bias in any category (haematological malignancies: t = 2.16, p = 0.052; neural tumors: t = -0.14, p = 0.891; other solid tumors: t = 1.54, p = 0.151) (see **eTable 2**, **eFigure Publication bias 4–6** for details).

In addition, the cumulative meta-analysis indicated that evidence supporting an increased risk of haematological malignancies (**Figure 3A**) and neural tumors (**Figure 3B**) among children conceived via ART emerged in 2022, with combined RR values fluctuating between 1.02 to 2.25 and 1.04 to 1.85, respectively. Since 2022, there have been no substantial changes in the direction or magnitude of these risk associations, which have remained stable. In contrast, evidence of risk associations between ART and other solid tumors (**Figure 3C**), which emerged in 2013, ranged from 0.74 to 2.28 for the combined RR. Data from 2018 further reinforced the strength of this risk association. Subsequent studies increased the precision of the point estimates but did not alter the direction or magnitude of the association.

### ART and the risk of specific cancers

**Figure 4:**
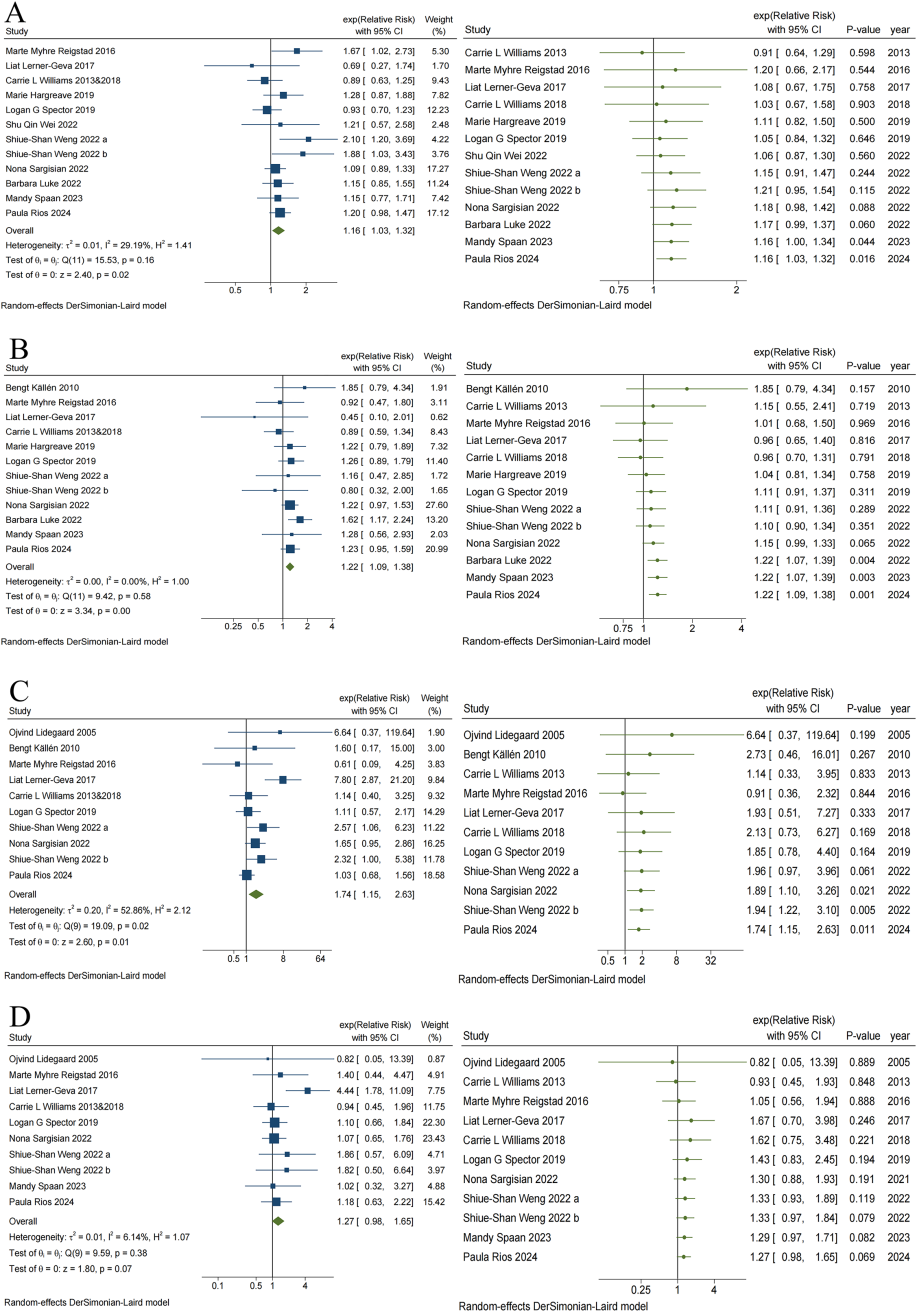
Traditional and Cumulative of Risk of Leukemia **(A)**, CNS tumors **(B)**, Retinoblastoma **(C)** and Renal tumors **(D)** in children conceived via ART.

**eTable 2** summarizes the associations of risk between ART and specific childhood cancers. Six specific cancers exhibited a significantly increased risk, including leukemia (RR = 1.16, 95% CI, 1.03–1.32) (**Figure 4A**), CNS tumors (RR = 1.22, 95% CI, 1.09–1.38) (**Figure 4B**), retinoblastoma (RR = 1.74, 95% CI, 1.15–2.63) (**Figure 4C**), hepatic tumors (RR = 2.73, 95% CI, 1.90–3.91), bone tumors and extraosseous sarcomas (RR = 1.62, 95% CI, 1.26–2.07), and epithelial tumors and melanoma (RR = 1.67, 95% CI, 1.22–2.29). Additionally, there were also seven specific cancers for which the risk was not significantly associated with ART, including lymphoma (RR = 1.12, 95% CI, 0.90–1.39), ALL (RR = 1.14, 95% CI, 0.95–1.36), AML (RR = 1.25, 95% CI, 0.72–2.18), peripheral nervous cell tumors (RR = 1.01, 95% CI, 0.79–1.29), renal tumors (RR = 1.27, 95% CI, 0.98–1.65) (**Figure 4D**), germ cell tumors (RR = 0.60, 95% CI, 0.25–1.42), and embryonal tumors (RR = 1.14, 95% CI, 0.99–1.32). Heterogeneity was observed solely in the study of retinoblastoma (I² = 52.86%, p = 0.02), suggesting that different types of cancer might significantly influence heterogeneity. Visual inspection of the funnel plot revealed symmetry, and no publication bias was detected via Egger’s test (p > 0.05), with the exception of germ cell tumors (t = -5.23, p = 0.035) (**eFigure Forest Plot 3–11**, **eFigure Publication bias 7–19**).

The results of the cumulative meta-analysis on ART and leukemia are presented in **Figure 4A**. Evidence indicating a risk of leukemia in children conceived via ART first emerged in 2024, with combined RR values ranging from 0.91 to 1.21. Additionally, evidence indicating the risk of CNS tumors (**Figure 4B**) and retinoblastoma (**Figure 4C**) in children conceived via ART first emerged in 2022, with combined RR values ranging from 0.96 to 1.85 and 0.91 to 6.64, respectively. No significant changes in direction, estimation, or precision were observed in the cumulative meta-analysis of these three cancer outcomes with subsequent additions to the study data. However, the cumulative meta-analysis of ART and renal tumor risk (**Figure 4D**) indicates that the effect of ART on renal tumors continues to be insignificant, despite the addition of new data over time.

### Subgroup analyses

**Figure 5:**
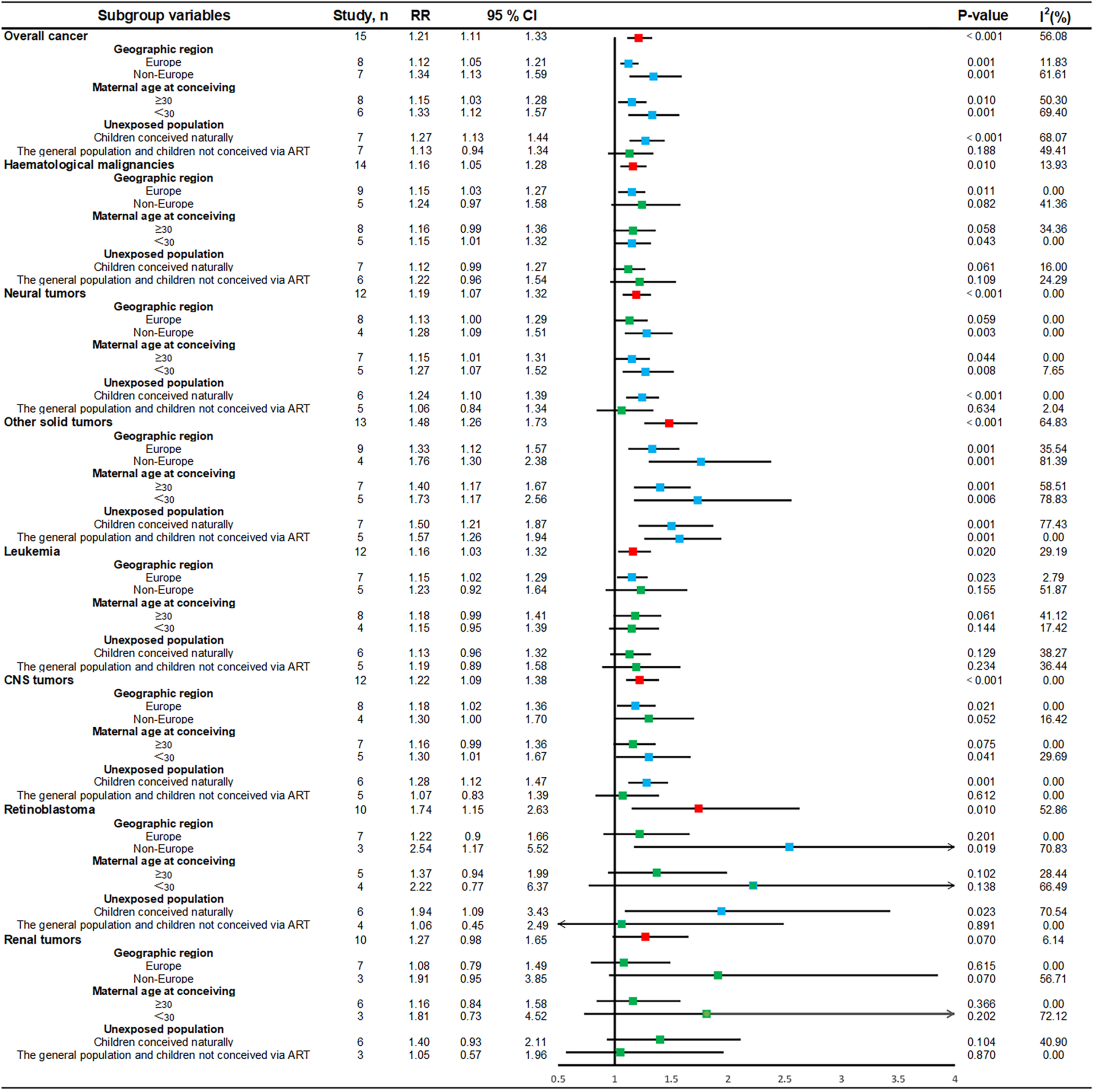
Subgroup forest plot of the association between ART and childhood cancer risk. CI, confidence interval; rr, relative risk; parallel lines, represent confidence intervals for individual effect sizes; vertical black line, invalid line of binary classification variable (1.00); red box, summary value of effect sizes for each type of cancer; green box, estimates of effect sizes that are currently not statistically significant; blue box, estimates of effect sizes that are statistically significant; I^2^, heterogeneity between studies within subgroups; p-value: p < 0.05 indicates statistical significance.

In this study, subgroup stratification was carried out for overall cancers, haematological malignancies, neural tumors, other solid tumors, leukemia, CNS tumors, retinoblastoma, and renal tumors among children conceived via ART (**eTable 4**, **Figure 5**, and **eFigure Subgroup Analysis Forest Plot 1–24**).

Stratified analyses by geographic region revealed a trend toward a significantly lower cancer risk for studies conducted in European countries, whereas non-European countries presented a significantly higher cancer risk ratio for overall cancers (RR = 1.34, 95% CI, 1.13–1.59), neural tumors (RR = 1.28, 95% CI, 1.09–1.51), other solid tumors (RR = 1.76, 95% CI, 1.30–2.38), and retinoblastoma (RR = 2.54, 95% CI, 1.17–5.52). Stratified analysis by maternal age at conceiving indicated a trend towards a reduced risk association between ART and childhood cancer when maternal age was ≥30 years, such as overall cancer (RR = 1.15, 95% CI, 1.03–1.28), neural tumors (RR = 1.15, 95% CI, 1.01–1.31), and other solid tumors (RR = 1.40, 95% CI, 1.17–1.67). In contrast, the risk associations tended to increase at maternal age at conceiving less than 30 years, as was observed for overall cancers (RR = 1.33, 95% CI, 1.12–1.57), neural tumors (RR = 1.27, 95% CI, 1.07–1.52), other solid tumors (RR = 1.73, 95% CI, 1.17–2.56), and CNS tumors (RR = 1.30, 95% CI, 1.01–1.67). Stratified by the unexposed population, there was a significantly increased risk ratio of cancer, such as overall cancer (RR = 1.27, 95% CI, 1.13–1.44), neural tumors (RR = 1.24, 95% CI, 1.10–1.39), other solid tumors (RR = 1.50, 95% CI, 1.21–1.87), CNS tumors (RR = 1.28, 95% CI, 1.12–1.47), and retinoblastoma (RR = 1.94, 95% CI, 1.09–3.43), and an increased risk of other solid tumors (RR = 1.57, 95% CI, 1.26–1.94), among children conceived via ART compared with the general population or children not conceived via ART.

Specific cancers often show a potentially positive associated risk in various types of stratified analyses; however, the rarity of these cancers makes conducting actual studies more challenging. In this regard, we cannot exclude the possibility that the elevated risk significance may not be detectable due to limited statistical power and an insufficient number of studies.

### IVF and overall risk of cancer

This meta-analysis did not find conclusive evidence for an association between IVF and childhood cancer risk (RR = 1.28, 95% CI, 0.98–1.66) (**eFigure Specific types of ART Forest Plot 1**). Importantly, owing to high heterogeneity (I² = 81.14%, p < 0.001), which was significantly reduced (I² = 45.49%, p = 0.14) after we considered the confounding of small-sample studies and excluded the study with the lowest number of exposures with an IVF exposure of 4,324 (51), the combined effect sizes were (RR = 1.13, 95% CI, 0.96–1.32) (**eFigure Specific types of ART Forest Plot 2**), and conclusions remained unchanged. Both before and after the exclusion of studies, visual funnel plots remained symmetrical, and Egger’s test did not reveal publication bias (all p > 0.05) (**eFigure Publication bias 20–21**).

### Sensitivity analysis

In a sensitivity analysis, this study evaluated the impact of various exclusion criteria on overall cancer risk estimates for children conceived via ART (**eFigure sensitivity analysis 2–3**). The results remained largely unchanged by the exclusion of either the five studies with differing confounder adjustments or the four studies that adjusted for ART exposure. Additionally, not a single study influenced the risk outcomes for overall cancer, haematological malignancies, neural tumors, other solid tumors, or specific cancers among children conceived via ART, confirming the robustness of the results (**eFigure sensitivity analysis 4-14**).

### Evidence appraisal using GRADE

In contrast to randomized controlled studies, observational studies are usually graded up or down from Grade C due to deficiencies in study design. For detailed evaluation procedures, see **etable 5**. Most of the results of the current study had an evidence grade of C, especially retinoblastoma, hepatic tumors, bone tumors and extraosseous sarcomas, which had a grade of B. Overall, the GRADE scores indicate that the level of evidence for the results of the current study is of high quality among observational studies and can effectively support the risk association between ART and childhood cancer.

## Discussion

To our knowledge, this is the largest and most comprehensive meta-analysis to explore the association between ART and childhood cancer risk, utilizing a large sample from observational studies. This meta-analysis, which involved 30 million children, demonstrated that ART was associated with an increased risk of overall cancer in children (RR = 1.21, 95% CI, 1.11–1.33) compared with natural conception, the general population, children not conceived via ART, or the unexposed population with low fertility but natural conception. Specifically, the risks for hematological malignancies (RR = 1.16, 95% CI, 1.05–1.28), neural tumors (RR = 1.19, 95% CI, 1.07–1.32), other solid tumors (RR = 1.48, 95% CI, 1.26–1.73), and certain specific cancers including leukemia, CNS tumors, retinoblastoma, hepatic tumors, bone tumors and extraosseous sarcomas, and epithelial tumors and melanomas, were significantly elevated. In addition, the findings suggested that different types of specific cancers might significantly influence the heterogeneity of the primary outcome. A further outcome of our research, a cumulative meta-analysis based on time lapses, revealed initial associations between ART and other solid tumors as early as 2013, with new cancer type risks emerging in 2022. Although subsequent studies have refined our estimates, no significant shifts in the direction or magnitude of risk associations have been observed, indicating stable outcomes. The data consistently support a robust link between ART and childhood cancer risk. However, we cannot rule out potential confounders such as the effects of ART and underlying parental infertility, which may contribute to increased cancer risk in offspring. Information on procedures such as IVF and ICSI (55), along with pertinent time-varying covariates during treatment (56), will aid in further clarifying the mechanisms underlying this association. Furthermore, this association remained consistent across subgroup analyses involving geographic regions, maternal age at conceiving, and populations of unexposed groups. Notably, women younger than 30 years who used ART might have had a higher risk ratio of cancer in their offspring (RR _age<30_ = 1.33, RR _age≥30_ = 1.15) highlighting the importance of promoting natural conception among younger women. These findings provide valuable insights for future research, clinical practice, and advice for couples considering ART.

Our results contradict those of a 2019 meta-analysis by Gilboa et al. (24), which reported no significant association between ART and childhood cancer risk (RR = 0.99; 95% CI, 0.85–1.15). This discrepancy may stem from Gilboa’s inclusion of data from 2018 case-control studies (57), potentially introducing bias into the pooled results. Conversely, Wang et al.’s (16) meta-analysis of observational studies from the same year, which explored fertility treatments and childhood cancer risk, aligned closely with our findings, with RRs for overall cancer, haematological malignancies, and other solid tumors in offspring of 1.16, 1.39, and 1.57, respectively. Nevertheless, their study concluded that the association between ART and the risk of neural tumors was not significant (RR = 1.15, 95% CI, 0.89–1.47), a finding that contradicts our conclusions. This discrepancy could be attributed to the inclusion of inconsistent study types and a greater number of small cohort studies (17, 31, 58, 59). In contrast, our study utilized more comprehensive and representative data, comprising 1,091,652 children conceived via ART and 1,789 childhood cancer cases, nearly threefold the number included in the 29 observational studies, which featured 327,884 children conceived through fertility treatments and 578 childhood cancer cases. In the 2020 study by Zhang et al. (25), which explored various fertility treatments and their risk for offspring cancer, our IVF findings aligned with their conclusions, revealing no evidence of an increased childhood cancer risk associated with IVF. Considering the scarcity of research on various ART types and their associations with childhood cancer risk, along with the very low incidence of cancer, the inclusion of numerous studies with small sample sizes and zero cancer cases in the exposure group in meta-analyses can exacerbate confounding issues, potentially leading to unreliable outcomes (60). Therefore, we advocate the inclusion of large studies to more accurately determine the actual risk association between ART and childhood cancer.

Despite pooling and analyzing data from a large sample of studies, we encountered the same issues as those reported by Wang et al. It remains unclear whether factors related to the ART implementation process or underlying parental fertility issues contribute to the increased risk of childhood cancers. The mechanisms underlying ART’s increased risk of childhood cancers remain largely unproven, with current hypotheses suggesting epigenetic disorders as a potentially influential pathway (61–63). Dynamic epigenetic regulation of gene imprinting, governed by both DNA methylation-dependent and DNA methylation-independent mechanisms, dictates the expression of specific genes on the basis of parental origin (64). Recent studies (65, 66) have established a connection between abnormal gene imprinting and the onset of childhood cancers, including retinoblastoma and neuroblastoma. Studies (67, 68) indicate that ART encompasses various stages of conception, ranging from gamete production stimulation to embryo *in vitro* culture, which includes processes such as ovarian stimulation, *in vitro* oocyte maturation, and gamete or embryo cryopreservation. Each step of the ART process has the potential to disrupt the normal genetic imprinting process, thereby increasing the risk of adverse pregnancy and neonatal outcomes (7, 69). Research conducted by Luke et al. (10) identified an increased cancer risk in children conceived via ART as well as their siblings who were not, suggesting that shared genetic or environmental factors may influence cancer susceptibility. In addition, infertility itself might increase epigenetic risk in children (70). Research (71) suggested that children born to mothers facing fertility challenges are at increased risk of developing cancer during childhood and early adulthood. Moreover, the largest cohort study in Asia, conducted by Weng et al. (11), indicated that this increased cancer risk may stem more from ART than from underlying fertility issues.

Existing studies have demonstrated heterogeneous results for the association between ART and childhood cancer risk. Both this study and the meta-analysis by Wang et al. indicated that the association of some cancer types with ART failed to reach statistical significance (72), potentially due to the low incidence of rare tumors leading to insufficient statistical power. However, several cohort studies have identified potential trends in risk for specific tumor types. A large cohort study in the United Kingdom, with a follow-up period of up to 17 years, observed no increase in overall cancer risk in ART-conceived children, but noted an increased risk of hepatoblastoma and rhabdomyosarcoma (18). This result is in alignment with the observed trend of increased risk of liver tumors in the US cohort (48). Conversely, the study across four Nordic countries highlighted an increased risk of central nervous system tumors and malignant epithelial tumors (73). Importantly, data from the Danish 10 million cohort revealed an elevated risk of leukemia, sympathetic nervous system tumors, and epithelial tumors and melanoma in FET-conceived children compared to naturally-conceived children, although these associations must be interpreted with caution due to the limited number of cases (n<5) (27, 47). Notably, the association of leukemia, the most common malignancy in children, with ART has demonstrated a relatively stable trend of elevated risk in multicenter studies (19, 20). The variability of the available evidence may be attributed to (1) confounding effects of underlying infertility and its associated pathologic states (2), biological differences in various ART technology options (e.g., IVF, FET), and (3) disparities in the completeness of cancer registry systems across geographic regions. Future studies should establish mega-sample cohorts through international multicenter collaborations and implement uniform exposure classification criteria to elucidate the causal associations and potential biological mechanisms between specific cancer types and ART.

We recommend cross-linking future international (non-national) registries that report outcomes after ART treatment with cancer registries. Additionally, these registries should record a specific set of variables associated with increased cancer risk, such as type of infertility, duration of infertility, mother’s age at conception, pregnancy cycle, primary versus secondary infertility, male infertility, parental smoking status, mother’s BMI, fetal growth/birth weight, and other relevant factors. To draw definitive conclusions, reliable databases are needed to avoid the risk of false or premature alarms, which can have detrimental consequences in couples’ counseling.

### Strengths and limitations

The key strengths of this evaluation include the following (1): Given the low prevalence of childhood cancer, the inclusion of a large dataset comprising 30 million childhood participants, 50,000 of whom had childhood cancer, enhances the statistical testing capabilities. This approach reduces potential confounders and minimizes small-sample bias, enabling more precise assessment of risk associations between ART and childhood cancer (2). All included studies are high-quality cohort studies rigorously assessed for their methodology, with risk of bias assessments and sensitivity analyses ensuring the credibility and robustness of findings (3). Time-cumulative meta-analysis was used to dynamically assess the trend between ART and childhood cancer risk, with consistent results confirming the positive risk association (4). Childhood cancers often develop before age 4 (74). A key strength of this study is the average follow-up period of 7.5 years, which enhances our ability to observe trends in overall and specific childhood cancer risks (5). The study explored various subgroup levels and ART types. Interestingly, the association with cancer risk in children conceived via ART was more pronounced when the mother was under 30 years of age at delivery, highlighting the importance of hierarchical analysis to complement the results. Overall, the results of this study are more comprehensive and robust than those of previous studies with smaller samples.

While the current meta-analysis has certain limitations, these limitations are primarily intrinsic to the individual studies included rather than to the meta-analytical methodology itself (1). The absence of specific ART-related details such as ovarian stimulation regimens and the limited number of unexposed people with low fertility but natural reproduction in the studies included in the current meta-analysis may interfere with an accurate assessment of the association between ART and the risk of cancer outcomes (2). The included studies were all cohort designs, reducing selection bias and categorization errors, but were also prone to missing-follow-up bias. The absence of case-control and cross-sectional studies might affect the completeness and certainty of the results (3). Additionally, the observational study design has deficiencies and requires further improvement. For example, controlling for concordance in fertility levels between exposed and non-exposed populations is essential to fully explore the risk association between ART and childhood cancer (4). We acknowledged that although all the studies included in this analysis were large cohort studies, the number of cases involving certain specific rare cancers was still small. This limitation affected the statistical precision of these estimates, and therefore caution needs to be exercised in interpreting the results, particularly with data on specific types of cancers (5). The associations between specific ART and various childhood cancers could not be thoroughly investigated due to the scarcity of extensive studies that uniformly reported on individual ART and particular types of cancer.

## Conclusions

Our meta-analysis, which involved high-quality observational studies with large samples, revealed a significant association between ART and increased risks of childhood cancer overall, haematological malignancies, neural tumors, other solid tumors, and six specific cancers. The results of the cumulative meta-analysis indicated that the direction and magnitude of the effects on these outcomes were largely consistent over time. These findings suggest that infertile couples considering ART should be aware of the potential increased cancer risk to their children.

## Data Availability

The original contributions presented in the study are included in the article/**Supplementary Material**. Further inquiries can be directed to the corresponding author/s.
