# Supplementary material for: Cumulative temporal association between assisted reproductive technology and childhood cancer: a systematic review and meta-analysis of observational studies
Source: Front Oncol. 2025 May 20;15:1555420. doi: 10.3389/fonc.2025.1555420 (PMC12129972; doi:10.3389/fonc.2025.1555420)
Supplement: Supplementary file 2 [file Supplementaryfile1.docx]

Supplementary catalogue

[Search strategies 1](#_Toc196657740)

[Research data Harmonization 3](#_Toc196657741)

[eFigure Forest Plot 1: Risk of Overall cancer in children conceived via ART (excluding 5 studies with differences in adjustment for confounders). 4](#_Toc196657742)

[eFigure Forest Plot 2: Risk of Overall cancer in children conceived via ART (excluding 4 studies of exposure substitution). 5](#_Toc196657743)

[eFigure Forest Plot 3: Risk of Lymphoma in children conceived via ART. 6](#_Toc196657744)

[eFigure Forest Plot 4: Risk of Acute lymphoblastic leukemia in children conceived via ART. 7](#_Toc196657745)

[eFigure Forest Plot 5: Risk of Acute myelocytic leukemia in children conceived via ART. 7](#_Toc196657746)

[eFigure Forest Plot 6: Risk of Peripheral nerve cell tumors in children conceived via ART. 8](#_Toc196657747)

[eFigure Forest Plot 7: Risk of Hepatic tumors in children conceived via ART. 9](#_Toc196657748)

[eFigure Forest Plot 8: Risk of Bone tumors and extraosseous sarcomas in children conceived via ART. 10](#_Toc196657749)

[eFigure Forest Plot 9: Risk of Germ cell tumors in children conceived via ART. 11](#_Toc196657750)

[eFigure Forest Plot 10: Risk of Embryonal tumors in children conceived via ART. 12](#_Toc196657751)

[eFigure Forest Plot 11: Risk of Epithelial tumors and melanoma in children conceived via ART. 13](#_Toc196657752)

[eFigure Subgroup analysis Forest Plot 1: Regional subgroup analysis of Overall cancer among children conceived via ART. 14](#_Toc196657753)

[eFigure Subgroup analysis Forest Plot 2: Maternal gestational age subgroup analysis of Overall cancer among children conceived by ART. 15](#_Toc196657754)

[eFigure Subgroup analysis Forest Plot 3: Unexposed population subgroup analysis of Overall cancer among children conceived by ART. 16](#_Toc196657755)

[eFigure Subgroup analysis Forest Plot 4: Regional subgroup analysis of Haematological malignancies among children conceived via ART. 17](#_Toc196657756)

[eFigure Subgroup analysis Forest Plot 5: Maternal gestational age subgroup analysis of Haematological malignancies among children conceived via ART. 18](#_Toc196657757)

[eFigure Subgroup analysis Forest Plot 6: Unexposed population subgroup analysis of Haematological malignancies among children conceived via ART. 19](#_Toc196657758)

[eFigure Subgroup analysis Forest Plot 7: Regional subgroup analysis of Neural tumors among children conceived via ART. 20](#_Toc196657759)

[eFigure Subgroup analysis Forest Plot 8: Maternal gestational age subgroup analysis of Neural tumors among children conceived via ART. 21](#_Toc196657760)

[eFigure Subgroup analysis Forest Plot 9: Unexposed population subgroup analysis of Neural tumors among children conceived via ART. 22](#_Toc196657761)

[eFigure Subgroup analysis Forest Plot 10: Regional subgroup analysis of Other solid tumors among children conceived via ART. 23](#_Toc196657762)

[eFigure Subgroup analysis Forest Plot 11: Maternal gestational age subgroup analysis of Other solid tumors among children conceived via ART. 24](#_Toc196657763)

[eFigure Subgroup analysis Forest Plot 12: Unexposed population subgroup analysis of Other solid tumors among children conceived via ART. 25](#_Toc196657764)

[eFigure Subgroup analysis Forest Plot 13: Regional subgroup analysis of Leukemia among children conceived via ART. 26](#_Toc196657765)

[eFigure Subgroup analysis Forest Plot 14: Maternal gestational age subgroup analysis of Leukemia among children conceived via ART. 27](#_Toc196657766)

[eFigure Subgroup analysis Forest Plot 15: Unexposed population subgroup analysis of Leukemia among children conceived via ART. 28](#_Toc196657767)

[eFigure Subgroup analysis Forest Plot 16: Regional subgroup analysis of CNS tumors among children conceived via ART. 29](#_Toc196657768)

[eFigure Subgroup analysis Forest Plot 17: Maternal gestational age subgroup analysis of CNS tumors among children conceived via ART. 30](#_Toc196657769)

[eFigure Subgroup analysis Forest Plot 18: Unexposed population subgroup analysis of CNS tumors among children conceived via ART. 31](#_Toc196657770)

[eFigure Subgroup analysis Forest Plot 19: Regional subgroup analysis of Retinoblastoma among children conceived via ART. 32](#_Toc196657771)

[eFigure Subgroup analysis Forest Plot 20: Maternal gestational age subgroup analysis of Retinoblastoma among children conceived via ART. 33](#_Toc196657772)

[eFigure Subgroup analysis Forest Plot 21: Unexposed population subgroup analysis of Retinoblastoma among children conceived via ART. 34](#_Toc196657773)

[eFigure Subgroup analysis Forest Plot 22: Regional subgroup analysis of Renal tumors among children conceived via ART. 35](#_Toc196657774)

[eFigure Subgroup analysis Forest Plot 23: Maternal gestational age subgroup analysis of Renal tumors among children conceived via ART. 36](#_Toc196657775)

[eFigure Subgroup analysis Forest Plot 24: Unexposed population subgroup analysis of Renal tumors among children conceived via ART. 37](#_Toc196657776)

[eFigure Specific types of ART Forest Plot 1: Overall cancer risk in children conceived through In vitro fertilization. 38](#_Toc196657777)

[eFigure Specific types of ART Forest Plot 2: Overall cancer risk in children conceived through In vitro fertilization (excluding Tamar Wainstock 2017). 38](#_Toc196657778)

[eFigure sensitivity analysis 1: Sensitivity analysis of overall cancer risk in children conceived via ART. 39](#_Toc196657779)

[eFigure sensitivity analysis 2: Sensitivity analysis of overall cancer risk in children conceived via ART (excluding 5 studies with differences in adjustment for confounders). 40](#_Toc196657780)

[eFigure sensitivity analysis 3: Sensitivity analysis of overall cancer risk in children conceived via ART (excluding 4 studies of exposure substitution). 41](#_Toc196657781)

[eFigure sensitivity analysis 4: Sensitivity analysis of Haematological malignancies risk in children conceived via ART. 42](#_Toc196657782)

[eFigure sensitivity analysis 5: Sensitivity analysis of Neural tumors risk in children conceived via ART. 43](#_Toc196657783)

[eFigure sensitivity analysis 6: Sensitivity analysis of Other solid tumors risk in children conceived via ART. 44](#_Toc196657784)

[eFigure sensitivity analysis 7: Sensitivity analysis of Leukemia risk in children conceived via ART. 45](#_Toc196657785)

[eFigure sensitivity analysis 8: Sensitivity analysis of Lymphoma risk in children conceived via ART. 46](#_Toc196657786)

[eFigure sensitivity analysis 9: Sensitivity analysis of CNS tumors risk in children conceived via ART. 47](#_Toc196657787)

[eFigure sensitivity analysis 10: Sensitivity analysis of Peripheral Nervous Cell tumors risk in children conceived via ART. 48](#_Toc196657788)

[eFigure sensitivity analysis 11: Sensitivity analysis of Retinoblastoma risk in children conceived via ART. 49](#_Toc196657789)

[eFigure sensitivity analysis 12: Sensitivity analysis of Hepatic tumors risk in children conceived via ART. 50](#_Toc196657790)

[eFigure sensitivity analysis 13: Sensitivity analysis of Renal tumors risk in children conceived via ART. 51](#_Toc196657791)

[eFigure sensitivity analysis 14: Sensitivity analysis of Bone tumors and extraosseous sarcomas risk in children conceived via ART. 52](#_Toc196657792)

[eFigure Publication bias 1: Funnel plot and Egger regression test for overall cancer risk in children conceived via ART. 53](#_Toc196657793)

[eFigure Publication bias 2: Funnel plot and Egger regression test for overall cancer risk in children conceived via ART (excluding 5 studies with differences in adjustment for confounders). 54](#_Toc196657794)

[eFigure Publication bias 3: Funnel plot and Egger regression test for overall cancer risk in children conceived via ART (excluding 4 studies of exposure substitution). 55](#_Toc196657795)

[eFigure Publication bias 4: Funnel plot and Egger regression test for Haematological malignancies risk in children conceived via ART. 56](#_Toc196657796)

[eFigure Publication bias 5: Funnel plot and Egger regression test for Neural tumors risk in children conceived via ART. 57](#_Toc196657797)

[eFigure Publication bias 6: Funnel plot and Egger regression test for Other solid tumors risk in children conceived via ART. 58](#_Toc196657798)

[eFigure Publication bias 7: Funnel plot and Egger regression test for Leukemia risk in children conceived via ART. 59](#_Toc196657799)

[eFigure Publication bias 8: Funnel plot and Egger regression test for Lymphoma risk in children conceived via ART. 60](#_Toc196657800)

[eFigure Publication bias 9: Funnel plot and Egger regression test for ALL risk in children conceived via ART. 61](#_Toc196657801)

[eFigure Publication bias 10: Funnel plot and Egger regression test for AML risk in children conceived via ART. 62](#_Toc196657802)

[eFigure Publication bias 11: Funnel plot and Egger regression test for CNS tumors risk in children conceived via ART. 63](#_Toc196657803)

[eFigure Publication bias 12: Funnel plot and Egger regression test for Peripheral Nervous Cell tumors risk in children conceived via ART. 64](#_Toc196657804)

[eFigure Publication bias 13: Funnel plot and Egger regression test for Retinoblastoma risk in children conceived via ART. 65](#_Toc196657805)

[eFigure Publication bias 14: Funnel plot and Egger regression test for Hepatic tumors risk in children conceived via ART. 66](#_Toc196657806)

[eFigure Publication bias 15: Funnel plot and Egger regression test for Renal tumors risk in children conceived via ART. 67](#_Toc196657807)

[eFigure Publication bias 16: Funnel plot and Egger regression test for Bone tumors and extraosseous sarcomas risk in children conceived via ART. 68](#_Toc196657808)

[eFigure Publication bias 17: Funnel plot and Egger regression test for Germ cell tumors risk in children conceived via ART. 69](#_Toc196657809)

[eFigure Publication bias 18: Funnel plot and Egger regression test for Embryonal tumors risk in children conceived via ART. 70](#_Toc196657810)

[eFigure Publication bias 19: Funnel plot and Egger regression test for Epithelial tumors and melanoma risk in children conceived via ART. 71](#_Toc196657811)

[eFigure Publication bias 20: Funnel plot and Egger regression test for Overall cancer risk in children conceived through In vitrofertilization. 72](#_Toc196657812)

[eFigure Publication bias 21: Funnel plot and Egger regression test for Overall cancer risk in children conceived through In vitrofertilization (excluding Tamar Wainstock 2017). 73](#_Toc196657813)

[eTable 1. List of excluded references and reasons for exclusion. 74](#_Toc196657814)

[eTable 2. Meta-analyses of risk estimates for cancers among children conceived via Assisted reproductive technology. 79](#_Toc196657815)

[eTable 3. NEWCASTLE - OTTAWA QUALITY ASSESSMENT SCALE (COHORT STUDIES) 81](#_Toc196657816)

[eTable 4. Subgroup analysis for risk of overall cancer, haematological malignancies, neural tumors, other solid tumors, Leukemia, CNS tumors, Retinoblastoma and Renal tumors among children conceived by ART. 82](#_Toc196657817)

[eTable 5. Grade Grading Details 84](#_Toc196657818)

# Search strategies

| **Database** | **Step** | **Terms** | **Results** |
| --- | --- | --- | --- |
| **PubMed** | 1 | (“Reproductive Techniques” [Mesh] OR “Reproductive Techniques”[Title/Abstract] OR “assisted reproductive”[Title/Abstract] OR “assisted conception”[Title/Abstract] OR “assisted reproduction”[Title/Abstract] OR “in vitro fertilization”[Title/Abstract] OR “test tube baby”[Title/Abstract] OR “intracytoplasmic sperm injection”[Title/Abstract] OR “artificial insemination”[Title/Abstract] OR “frozen-thawed embryo transfer”[Title/Abstract] OR “embryo transfer”[Title/Abstract] OR “intrauterine insemination”[Title/Abstract] OR “cervical canal insemination”[Title/Abstract] OR “embryo implantation”[Title/Abstract] OR “ART”[Title/Abstract] OR “AI”[Title/Abstract] OR “IVF”[Title/Abstract] OR “ICSI”[Title/Abstract] OR “FET”[Title/Abstract] OR “infertility treatment”[Title/Abstract] OR “fertility treatment”[Title/Abstract]) |  |
|  | 2 | (“Neoplasms/epidemiology”[Mesh] OR “Tumor”[Title/Abstract] OR “Tumors”[Title/Abstract] OR “Tumour”[Title/Abstract] OR “Tumours”[Title/Abstract] OR “cancer”[Title/Abstract] OR “cancers”[Title/Abstract] OR “Neoplasia”[Title/Abstract] OR “Neoplastic”[Title/Abstract] OR “Neoplasm”[Title/Abstract] OR “Neoplasms”[Title/Abstract] OR “carcinoma”[Title/Abstract] OR “malignancy”[Title/Abstract] OR “malignancies”[Title/Abstract] OR “malignant”[Title/Abstract] OR “Leukemia”[Mesh] OR “Myeloproliferative Disorders”[Mesh] OR “Myeloproliferative Diseases”[Mesh] OR “Lymphoma”[Mesh] OR “Central Nervous System Neoplasms”[Mesh] OR “Glioma”[Mesh] OR “Neuroectodermal Tumors, Primitive”[Mesh]OR “Neuroblastoma”[Mesh] OR “Retinoblastoma”[Mesh] OR “Kidney Neoplasms”[Mesh] OR “Liver Neoplasms”[Mesh] OR “Hepatoblastoma”[Mesh] OR “Bone Neoplasms”[Mesh] OR “Osteosarcoma”[Mesh] OR “Chondrosarcoma”[Mesh] OR “Sarcoma”[Mesh] OR “Adrenocortical Carcinoma”[Mesh] OR “Thyroid Neoplasms”[Mesh] OR “Nasopharyngeal Neoplasms”[Mesh] OR “Melanoma”[Mesh] OR “Neoplasms, Germ Cell and Embryonal”[Mesh] OR “Neoplasms, Gonadal Tissue”[Mesh]) |  |
|  | 3 | (“infant”[Mesh] OR “child”[Mesh] OR “adolescent”[Mesh] OR “pediatric”[Mesh] OR “paediatric”[Mesh] OR “children”[Mesh] OR “childhood”[Mesh] OR “offspring”[Mesh] OR “infant”[Title/Abstract] OR “child”[Title/Abstract] OR “adolescent”[Title/Abstract] OR “pediatric”[Title/Abstract] OR “paediatric”[Title/Abstract] OR “children”[Title/Abstract] OR “childhood”[Title/Abstract] OR “offspring”[Title/Abstract]) |  |
|  | 4 | #1 AND #2 AND #3 | **4,091** |
| **Embase** | 1 | 'Reproductive Techniques'/exp OR 'assisted therapy'/exp OR 'assisted conception'/exp OR 'assisted reproduction'/exp OR 'in vitro fertilization'/exp OR 'test tube baby'/exp OR 'intracytoplasmic sperm injection'/exp OR 'artificial insemination'/exp OR 'frozen-thawed embryo transfer'/exp OR 'embryo transfer'/exp OR 'intrauterine insemination'/exp OR 'cervical canal insemination'/exp OR 'embryo implantation'/exp OR 'infertility treatment'/exp OR 'fertility treatment'/exp OR 'Reproductive Techniques':ab,ti,kw OR 'assisted reproductive':ab,ti,kw OR 'assisted therapy':ab,ti,kw OR 'assisted conception':ab,ti,kw OR 'assisted reproduction':ab,ti,kw OR 'in vitro fertilization':ab,ti,kw OR 'test tube baby':ab,ti,kw OR 'intracytoplasmic sperm injection':ab,ti,kw OR 'artificial insemination':ab,ti,kw OR 'frozen-thawed embryo transfer':ab,ti,kw OR 'embryo transfer':ab,ti,kw OR 'intrauterine insemination':ab,ti,kw OR 'cervical canal insemination':ab,ti,kw OR 'embryo implantation':ab,ti,kw OR 'ART':ab,ti,kw OR 'AI':ab,ti,kw OR 'IVF':ab,ti,kw OR 'ICSI':ab,ti,kw OR 'FET':ab,ti,kw OR 'infertility treatment':ab,ti,kw OR 'fertility treatment':ab,ti,kw |  |
|  | 2 | 'neoplasm'/exp OR 'Neoplasm':ab,ti,kw OR 'Tumor':ab,ti,kw OR 'Tumors':ab,ti,kw OR 'Tumour':ab,ti,kw OR 'Tumours':ab,ti,kw OR 'cancer':ab,ti,kw OR 'cancers':ab,ti,kw OR 'Neoplasia':ab,ti,kw OR 'Neoplastic':ab,ti,kw OR 'Neoplasm':ab,ti,kw OR 'Neoplasms':ab,ti,kw OR 'carcinoma':ab,ti,kw OR 'malignancy':ab,ti,kw OR 'malignancies':ab,ti,kw OR 'malignant':ab,ti,kw OR 'Leukemia'/exp OR 'Myeloproliferative Disorders'/exp OR 'mixed myelodysplastic myeloproliferative disease'/exp OR 'Lymphoma'/exp OR 'Central Nervous System'/exp OR 'Neoplasms'/exp OR 'Glioma'/exp OR 'neuroectoderm tumor'/exp OR 'Neuroblastoma'/exp OR 'Retinoblastoma'/exp OR 'Kidney Neoplasms'/exp OR 'Liver Neoplasms'/exp OR 'Hepatoblastoma'/exp OR 'Bone Neoplasms'/exp OR 'Osteosarcoma'/exp OR 'Chondrosarcoma'/exp OR 'Sarcoma'/exp OR 'Adrenocortical Carcinoma'/exp OR 'Thyroid Neoplasms'/exp OR 'Nasopharyngeal Neoplasms'/exp OR 'Melanoma'/exp OR 'germ cell and embryonal neoplasms'/exp OR 'gonad tumor'/exp |  |
|  | 3 | 'infant'/exp OR 'child'/exp OR 'adolescent'/exp OR 'pediatric'/exp OR 'paediatric'/exp OR 'children'/exp OR 'childhood'/exp OR 'offspring'/exp OR 'infant':ab,ti,kw OR 'child':ab,ti,kw OR 'adolescent':ab,ti,kw OR 'pediatric':ab,ti,kw OR 'paediatric':ab,ti,kw OR 'children':ab,ti,kw OR 'childhood':ab,ti,kw OR 'offspring':ab,ti,kw |  |
|  | 4 | #1 AND #2 AND #3 | **9,619** |
| **Web of Science** | 1 | TS=("Reproductive Techniques" OR "Reproductive Techniques" OR "assisted reproductive" OR "assisted conception" OR "assisted reproduction" OR "in vitro fertilization" OR "test tube baby" OR "intracytoplasmic sperm injection" OR "artificial insemination" OR "frozen-thawed embryo transfer" OR "embryo transfer" OR "intrauterine insemination" OR "cervical canal insemination" OR "embryo implantation" OR "ART" OR "AI" OR "IVF" OR "ICSI" OR "FET" OR "infertility treatment" OR "fertility treatment") |  |
|  | 2 | TS=("Neoplasms" OR "Tumor" OR "Tumors" OR "Tumour" OR "Tumours" OR "cancer" OR "cancers" OR "Neoplasia" OR "Neoplastic" OR "Neoplasm" OR "Neoplasms" OR "carcinoma" OR "malignancy" OR "malignancies" OR "malignant" OR "Leukemia" OR "Myeloproliferative Disorders" OR "Myeloproliferative Diseases" OR "Lymphoma" OR "Central Nervous System" OR "Neoplasms" OR "Glioma" OR "Neuroectodermal Tumors, Primitive" OR "Neuroblastoma" OR "Retinoblastoma" OR "Kidney Neoplasms" OR "Liver Neoplasms" OR "Hepatoblastoma" OR "Bone Neoplasms" OR "Osteosarcoma" OR "Chondrosarcoma" OR "Sarcoma" OR "Adrenocortical Carcinoma" OR "Thyroid Neoplasms" OR "Nasopharyngeal Neoplasms" OR "Melanoma" OR "Neoplasms, Germ Cell and Embryonal" OR "Neoplasms, Gonadal Tissue") |  |
|  | 3 | TS=("infant" OR "child" OR "adolescent" OR "pediatric" OR "paediatric" OR "children" OR "childhood" OR "offspring") |  |
|  | 4 | #1 AND #2 AND #3 | **283** |
| **Cochrane Library** | 1 | MeSH descriptor: [Reproductive Techniques] explode all trees OR MeSH descriptor: [Fertilization in Vitro] explode all trees OR MeSH descriptor: [Sperm Injections, Intracytoplasmic] explode all trees OR MeSH descriptor: [Insemination, Artificial] explode all trees OR MeSH descriptor: [Embryo Implantation] explode all trees OR MeSH descriptor: [Embryo Transfer] explode all trees OR ("Reproductive Techniques" OR "Reproductive Techniques" OR "assisted reproductive" OR "assisted conception" OR "assisted reproduction" OR "in vitro fertilization" OR "test tube baby" OR "intracytoplasmic sperm injection" OR "artificial insemination" OR "frozen-thawed embryo transfer" OR "embryo transfer" OR "intrauterine insemination" OR "cervical canal insemination" OR "embryo implantation" OR "ART" OR "AI" OR "IVF" OR "ICSI" OR "FET" OR "infertility treatment" OR "fertility treatment"):ti,ab,kw |  |
|  | 2 | MeSH descriptor: [Neoplasms] explode all trees OR MeSH descriptor: [Leukemia] explode all trees OR MeSH descriptor: [Myeloproliferative Disorders] explode all trees OR MeSH descriptor: [Lymphoma] explode all trees OR MeSH descriptor: [Central Nervous System] explode all trees OR MeSH descriptor: [Neoplasms] explode all trees OR MeSH descriptor: [Glioma] explode all trees OR MeSH descriptor: [Neuroectodermal Tumors, Primitive] explode all trees OR MeSH descriptor: [Neuroblastoma] explode all trees OR MeSH descriptor: [Retinoblastoma] explode all trees OR MeSH descriptor: [Kidney Neoplasms] explode all trees OR MeSH descriptor: [Liver Neoplasms] explode all trees OR MeSH descriptor: [Hepatoblastoma] explode all trees OR MeSH descriptor: [Bone Neoplasms] explode all trees OR MeSH descriptor: [Osteosarcoma] explode all trees OR MeSH descriptor: [Sarcoma] explode all trees OR MeSH descriptor: [Adrenocortical Carcinoma] explode all trees OR MeSH descriptor: [Chondrosarcoma] explode all trees OR MeSH descriptor: [Thyroid Neoplasms] explode all trees OR MeSH descriptor: [Nasopharyngeal Neoplasms] explode all trees OR MeSH descriptor: [Melanoma] explode all trees OR MeSH descriptor: [Neoplasms, Germ Cell and Embryonal] explode all trees OR MeSH descriptor: [Neoplasms, Gonadal Tissue] explode all trees OR ("Neoplasms" OR "Tumor" OR "Tumors" OR "Tumour" OR "Tumours" OR "cancer" OR "cancers" OR "Neoplasia" OR "Neoplastic" OR "Neoplasm" OR "Neoplasms" OR "carcinoma" OR "malignancy" OR "malignancies" OR "malignant" OR "Leukemia" OR "Myeloproliferative Disorders" OR "Myeloproliferative Diseases" OR "Lymphoma" OR "Central Nervous System" OR "Neoplasms" OR "Glioma" OR "Neuroectodermal Tumors, Primitive" OR "Neuroblastoma" OR "Retinoblastoma" OR "Kidney Neoplasms" OR "Liver Neoplasms" OR "Hepatoblastoma" OR "Bone Neoplasms" OR "Osteosarcoma" OR "Chondrosarcoma" OR "Sarcoma" OR "Adrenocortical Carcinoma" OR "Thyroid Neoplasms" OR "Nasopharyngeal Neoplasms" OR "Melanoma" OR "Neoplasms, Germ Cell and Embryonal" OR "Neoplasms, Gonadal Tissue"):ti,ab,kw |  |
|  | 3 | MeSH descriptor: [Infant] explode all trees OR MeSH descriptor: [Child] explode all trees OR MeSH descriptor: [Adolescent] explode all trees OR MeSH descriptor: [Pediatrics] explode all trees OR ("infant" OR "child" OR "adolescent" OR "pediatric" OR "paediatric" OR "children" OR "childhood" OR "offspring"):ti,ab,kw |  |
|  | 4 | #1 AND #2 AND #3 | **312** |
| **Total** |  |  | **14,305** |

# **Research data Harmonization**

According to Wang et al.(1), for the treatment of data that report the risk of different types of cancers within the same category, the RRs of the same combined cancer category are calculated for pooled analyses in the same study via a fixed-effects model. For example, a study reporting risk estimates separately for diseases such as CNS tumors, various intracranial and spinal tumors, neuroblastoma, and other peripheral nervous cell tumors would consolidate these estimates into risk estimates for the neural tumors category.

For studies evaluating the associations between any ART, several types, or a single type of ART and the risk of childhood cancer, risk estimates for any ART were used for pooled analyses. If no outcomes were reported for any ART, risk estimates for several types of ART would be combined for analyses in the same study via fixed-effects models. For example, studies by Rios et al.(2) involving fresh embryo transfer (fresh ET), frozen ET (FET), artificial insemination (AI), and Luke et al.(3) involving ART-Autologous/Donor-Fresh/Thawed in relation to childhood cancer risks, their RRs were combined and pooled for the main analysis via the aforementioned methodology. Studies whose results were risk estimates for a single type of ART were directly included in the pooled analysis.

Additionally, two UK-based cohort studies, one involving children conceived via nondonor ART(4) and the other involving children conceived via donor ART(5), employed fixed effects models to calculate the combined RR for analysis.

1. Wang T, Chen L, Yang T, Wang L, Zhao L, Zhang S, et al. Cancer risk among children conceived by fertility treatment. International journal of cancer. 2019;144(12):3001-13.

2. Rios P, Herlemont P, Fauque P, Lacour B, Jouannet P, Weill A, et al. Medically Assisted Reproduction and Risk of Cancer Among Offspring. JAMA network open. 2024;7(5):e249429.

3. Luke B, Brown MB, Wantman E, Schymura MJ, Browne ML, Fisher SC, et al. The risks of birth defects and childhood cancer with conception by assisted reproductive technology. Human reproduction (Oxford, England). 2022;37(11):2672-89.

4. Williams CL, Bunch KJ, Stiller CA, Murphy MF, Botting BJ, Wallace WH, et al. Cancer risk among children born after assisted conception. The New England journal of medicine. 2013;369(19):1819-27.

5. Williams CL, Bunch KJ, Murphy MFG, Stiller CA, Botting BJ, Wallace WH, et al. Cancer risk in children born after donor ART. Human reproduction (Oxford, England). 2018;33(1):140-6.

# **eFigure Forest Plot 1:** Risk of Overall cancer in children conceived via ART (excluding 5 studies with differences in adjustment for confounders).

eFigure Forest Plot 2: Risk of Overall cancer in children conceived via ART (excluding 4 studies of exposure substitution).

# eFigure Forest Plot 3: Risk of Lymphoma in children conceived via ART.

eFigure Forest Plot 4: Risk of Acute lymphoblastic leukemia in children conceived via ART.

eFigure Forest Plot 5: Risk of Acute myelocytic leukemia in children conceived via ART.

# eFigure Forest Plot 6: Risk of Peripheral nerve cell tumors in children conceived via ART.

# eFigure Forest Plot 7: Risk of Hepatic tumors in children conceived via ART.

# eFigure Forest Plot 8: Risk of Bone tumors and extraosseous sarcomas in children conceived via ART.

eFigure Forest Plot 9: Risk of Germ cell tumors in children conceived via ART.

eFigure Forest Plot 10: Risk of Embryonal tumors in children conceived via ART.

# eFigure Forest Plot 11: Risk of Epithelial tumors and melanoma in children conceived via ART.

eFigure Subgroup analysis Forest Plot 1: Regional subgroup analysis of Overall cancer among children conceived via ART.

eFigure Subgroup analysis Forest Plot 2: Maternal gestational age subgroup analysis of Overall cancer among children conceived by ART.

eFigure Subgroup analysis Forest Plot 3: Unexposed population subgroup analysis of Overall cancer among children conceived by ART.

eFigure Subgroup analysis Forest Plot 4: Regional subgroup analysis of Haematological malignancies among children conceived via ART.

# **eFigure Subgroup analysis Forest Plot 5**: Maternal gestational age subgroup analysis of Haematological malignancies among children conceived via ART.


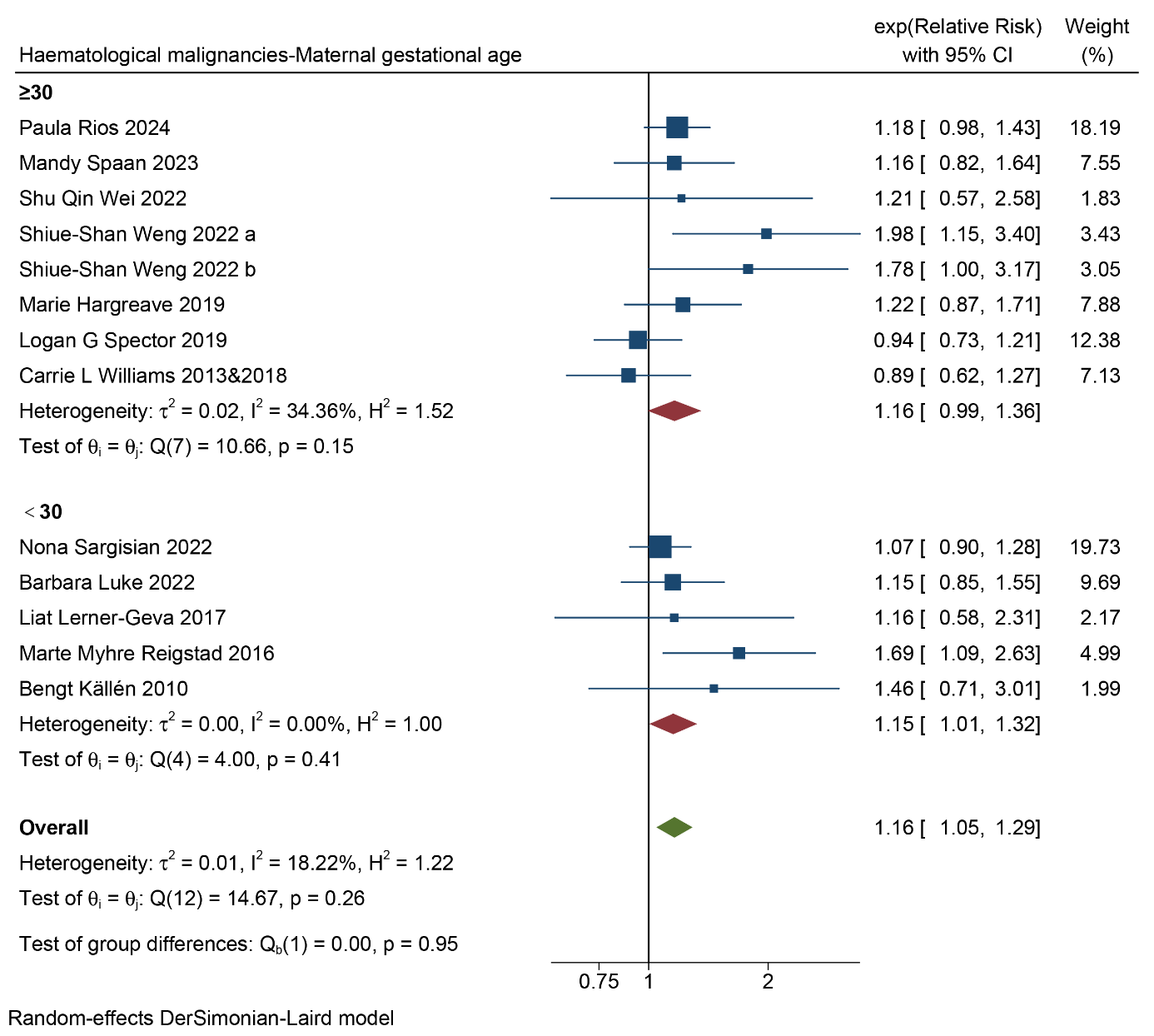


# eFigure Subgroup analysis Forest Plot 6: Unexposed population subgroup analysis of Haematological malignancies among children conceived via ART.

eFigure Subgroup analysis Forest Plot 7: Regional subgroup analysis of Neural tumors among children conceived via ART.

eFigure Subgroup analysis Forest Plot 8: Maternal gestational age subgroup analysis of Neural tumors among children conceived via ART.
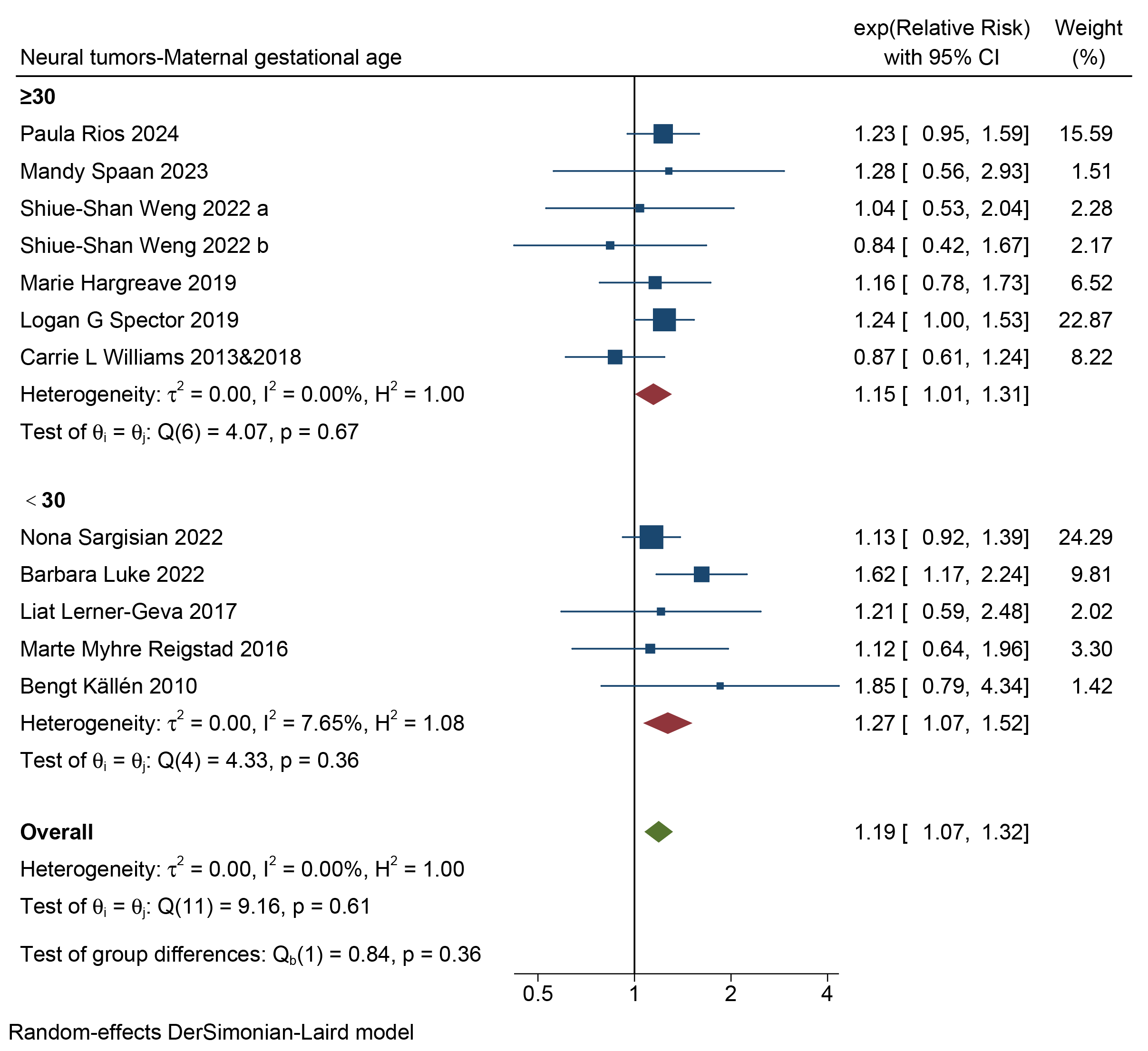


eFigure Subgroup analysis Forest Plot 9: Unexposed population subgroup analysis of Neural tumors among children conceived via ART.

eFigure Subgroup analysis Forest Plot 10: Regional subgroup analysis of Other solid tumors among children conceived via ART.

eFigure Subgroup analysis Forest Plot 11: Maternal gestational age subgroup analysis of Other solid tumors among children conceived via ART.

eFigure Subgroup analysis Forest Plot 12: Unexposed population subgroup analysis of Other solid tumors among children conceived via ART.

eFigure Subgroup analysis Forest Plot 13: Regional subgroup analysis of Leukemia among children conceived via ART.

eFigure Subgroup analysis Forest Plot 14: Maternal gestational age subgroup analysis of Leukemia among children conceived via ART.

eFigure Subgroup analysis Forest Plot 15: Unexposed population subgroup analysis of Leukemia among children conceived via ART.

eFigure Subgroup analysis Forest Plot 16: Regional subgroup analysis of CNS tumors among children conceived via ART.

eFigure Subgroup analysis Forest Plot 17: Maternal gestational age subgroup analysis of CNS tumors among children conceived via ART.

eFigure Subgroup analysis Forest Plot 18: Unexposed population subgroup analysis of CNS tumors among children conceived via ART.

eFigure Subgroup analysis Forest Plot 19: Regional subgroup analysis of Retinoblastoma among children conceived via ART.

eFigure Subgroup analysis Forest Plot 20: Maternal gestational age subgroup analysis of Retinoblastoma among children conceived via ART.

eFigure Subgroup analysis Forest Plot 21: Unexposed population subgroup analysis of Retinoblastoma among children conceived via ART.

eFigure Subgroup analysis Forest Plot 22: Regional subgroup analysis of Renal tumors among children conceived via ART.

eFigure Subgroup analysis Forest Plot 23: Maternal gestational age subgroup analysis of Renal tumors among children conceived via ART.

eFigure Subgroup analysis Forest Plot 24: Unexposed population subgroup analysis of Renal tumors among children conceived via ART.

eFigure Specific types of ART Forest Plot 1: Overall cancer risk in children conceived through In vitro fertilization.

# eFigure Specific types of ART Forest Plot 2: Overall cancer risk in children conceived through In vitro fertilization (excluding Tamar Wainstock 2017).

# eFigure sensitivity analysis 1: Sensitivity analysis of overall cancer risk in children conceived via ART.

# eFigure sensitivity analysis 2: Sensitivity analysis of overall cancer risk in children conceived via ART (excluding 5 studies with differences in adjustment for confounders).

# eFigure sensitivity analysis 3: Sensitivity analysis of overall cancer risk in children conceived via ART (excluding 4 studies of exposure substitution).

# eFigure sensitivity analysis 4: Sensitivity analysis of Haematological malignancies risk in children conceived via ART.

# eFigure sensitivity analysis 5: Sensitivity analysis of Neural tumors risk in children conceived via ART.

# eFigure sensitivity analysis 6: Sensitivity analysis of Other solid tumors risk in children conceived via ART.


eFigure sensitivity analysis 7: Sensitivity analysis of Leukemia risk in children conceived via ART.

# eFigure sensitivity analysis 8: Sensitivity analysis of Lymphoma risk in children conceived via ART.


eFigure sensitivity analysis 9: Sensitivity analysis of CNS tumors risk in children conceived via ART.

# eFigure sensitivity analysis 10: Sensitivity analysis of Peripheral Nervous Cell tumors risk in children conceived via ART.

eFigure sensitivity analysis 11: Sensitivity analysis of Retinoblastoma risk in children conceived via ART.


eFigure sensitivity analysis 12: Sensitivity analysis of Hepatic tumors risk in children conceived via ART.
eFigure sensitivity analysis 13: Sensitivity analysis of Renal tumors risk in children conceived via ART.
eFigure sensitivity analysis 14: Sensitivity analysis of Bone tumors and extraosseous sarcomas risk in children conceived via ART.

# eFigure Publication bias 1: Funnel plot and Egger regression test for overall cancer risk in children conceived via ART.


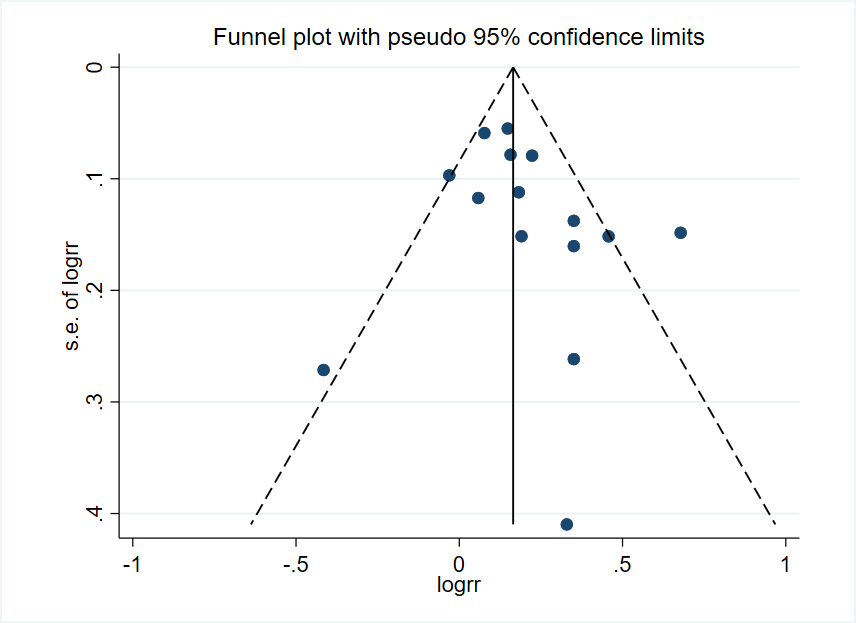

# eFigure Publication bias 2: Funnel plot and Egger regression test for overall cancer risk in children conceived via ART (excluding 5 studies with differences in adjustment for confounders).

# eFigure Publication bias 3: Funnel plot and Egger regression test for overall cancer risk in children conceived via ART (excluding 4 studies of exposure substitution).

# eFigure Publication bias 4: Funnel plot and Egger regression test for Haematological malignancies risk in children conceived via ART.

# eFigure Publication bias 5: Funnel plot and Egger regression test for Neural tumors risk in children conceived via ART.

# eFigure Publication bias 6: Funnel plot and Egger regression test for Other solid tumors risk in children conceived via ART.

# eFigure Publication bias 7: Funnel plot and Egger regression test for Leukemia risk in children conceived via ART.

# eFigure Publication bias 8: Funnel plot and Egger regression test for Lymphoma risk in children conceived via ART.

# eFigure Publication bias 9: Funnel plot and Egger regression test for ALL risk in children conceived via ART.

# eFigure Publication bias 10: Funnel plot and Egger regression test for AML risk in children conceived via ART.

# eFigure Publication bias 11: Funnel plot and Egger regression test for CNS tumors risk in children conceived via ART.

# eFigure Publication bias 12: Funnel plot and Egger regression test for Peripheral Nervous Cell tumors risk in children conceived via ART.

# eFigure Publication bias 13: Funnel plot and Egger regression test for Retinoblastoma risk in children conceived via ART.

# eFigure Publication bias 14: Funnel plot and Egger regression test for Hepatic tumors risk in children conceived via ART.

# eFigure Publication bias 15: Funnel plot and Egger regression test for Renal tumors risk in children conceived via ART.

# eFigure Publication bias 16: Funnel plot and Egger regression test for Bone tumors and extraosseous sarcomas risk in children conceived via ART.

# eFigure Publication bias 17: Funnel plot and Egger regression test for Germ cell tumors risk in children conceived via ART.

# eFigure Publication bias 18: Funnel plot and Egger regression test for Embryonal tumors risk in children conceived via ART.

eFigure Publication bias 19: Funnel plot and Egger regression test for Epithelial tumors and melanoma risk in children conceived via ART.

# eFigure Publication bias 20: Funnel plot and Egger regression test for Overall cancer risk in children conceived through In vitrofertilization.

# eFigure Publication bias 21: Funnel plot and Egger regression test for Overall cancer risk in children conceived through In vitrofertilization (excluding Tamar Wainstock 2017).

# eTable 1. List of excluded references and reasons for exclusion.

| **Study year** | **References** | **Reasons** |
| --- | --- | --- |
| Brian D Bradbury 2004 | 1. Bradbury BD, Jick H. In vitro fertilization and childhood retinoblastoma. Br J Clin Pharmacol. 2004 Aug;58(2):209-11. doi: 10.1111/j.1365-2125.2004.02109.x. | The number of children conceived via ART < 4000. |
| Jérémie Rudant 2013 | 1. Rudant J, Amigou A, Orsi L, Althaus T, Leverger G, Baruchel A, Bertrand Y, Nelken B, Plat G, Michel G, Sirvent N, Chastagner P, Ducassou S, Rialland X, Hémon D, Clavel J. Fertility treatments, congenital malformations, fetal loss, and childhood acute leukemia: the ESCALE study (SFCE). Pediatr Blood Cancer. 2013 Feb;60(2):301-8. doi: 10.1002/pbc.24192. Epub 2012 May 18. | The number of children conceived via ART < 4000. |
| P Doyle 1998 | 1. Doyle P, Bunch KJ, Beral V, Draper GJ. Cancer incidence in children conceived with assisted reproduction technology. Lancet. 1998 Aug 8;352(9126):452-3. doi: 10.1016/s0140-6736(05)79186-8. | Duplication. |
| T Bergh 1999 | 1. Bergh T, Ericson A, Hillensjö T, Nygren KG, Wennerholm UB. Deliveries and children born after in-vitro fertilisation in Sweden 1982-95: a retrospective cohort study. Lancet. 1999 Nov 6;354(9190):1579-85. doi: 10.1016/S0140-6736(99)04345-7. | Duplication. |
| L Lerner-Geva 2000 | 1. Lerner-Geva L, Toren A, Chetrit A, Modan B, Mandel M, Rechavi G, Dor J. The risk for cancer among children of women who underwent in vitro fertilization. Cancer. 2000 Jun 15;88(12):2845-7. doi: 10.1002/1097-0142(20000615)88:12<2845::aid-cncr26>3.0.co;2-e. | Duplication. |
| H Klip 2001 | 1. Klip H, Burger CW, de Kraker J, van Leeuwen FE; OMEGA-project group. Risk of cancer in the offspring of women who underwent ovarian stimulation for IVF. Hum Reprod. 2001 Nov;16(11):2451-8. doi: 10.1093/humrep/16.11.2451. | Duplication. |
| A Ericson 2002 | 1. Ericson A, Nygren KG, Olausson PO, Källén B. Hospital care utilization of infants born after IVF. Hum Reprod. 2002 Apr;17(4):929-32. doi: 10.1093/humrep/17.4.929. | Duplication. |
| Anja Pinborg 2004 | 1. Pinborg A, Loft A, Rasmussen S, Schmidt L, Langhoff-Roos J, Greisen G, Andersen AN. Neonatal outcome in a Danish national cohort of 3438 IVF/ICSI and 10,362 non-IVF/ICSI twins born between 1995 and 2000. Hum Reprod. 2004 Feb;19(2):435-41. doi: 10.1093/humrep/deh063. | Duplication. |
| Bengt Källén 2005 | 1. Källén B, Finnström O, Nygren KG, Olausson PO. In vitro fertilization in Sweden: child morbidity including cancer risk. Fertil Steril. 2005 Sep;84(3):605-10. doi: 10.1016/j.fertnstert.2005.03.035. | Duplication. |
| Nathalie Mallol-Mesnard 2008 | 1. Mallol-Mesnard N, Menegaux F, Lacour B, Hartmann O, Frappaz D, Doz F, Bertozzi AI, Chastagner P, Hémon D, Clavel J. Birth characteristics and childhood malignant central nervous sytem tumors: the ESCALE study (French Society for Childhood Cancer). Cancer Detect Prev. 2008;32(1):79-86. doi: 10.1016/j.cdp.2008.02.003. Epub 2008 Apr 8. | Duplication. |
| Anja Pinborg 2010 | 1. Pinborg A, Loft A, Aaris Henningsen AK, Rasmussen S, Andersen AN. Infant outcome of 957 singletons born after frozen embryo replacement: the Danish National Cohort Study 1995-2006. Fertil Steril. 2010 Sep;94(4):1320-1327. doi: 10.1016/j.fertnstert.2009.05.091. Epub 2009 Jul 31. | Duplication. |
| L Foix-L'Hélias 2012 | 1. Foix-L'Hélias L, Aerts I, Marchand L, Lumbroso-Le Rouic L, Gauthier-Villars M, Labrune P, Bouyer J, Doz F, Kaminski M. Are children born after infertility treatment at increased risk of retinoblastoma? Hum Reprod. 2012 Jul;27(7):2186-92. doi: 10.1093/humrep/des149. Epub 2012 May 15. | Duplication. |
| Karin Jerhamre Sundh 2014 | 1. Sundh KJ, Henningsen AK, Källen K, Bergh C, Romundstad LB, Gissler M, Pinborg A, Skjaerven R, Tiitinen A, Vassard D, Lannering B, Wennerholm UB. Cancer in children and young adults born after assisted reproductive technology: a Nordic cohort study from the Committee of Nordic ART and Safety (CoNARTaS). Hum Reprod. 2014 Sep;29(9):2050-7. doi: 10.1093/humrep/deu143. Epub 2014 Jul 2. | Duplication. |
| Tamar Wainstock 2017 | 1. Wainstock T, Walfisch A, Shoham-Vardi I, Segal I, Harlev A, Sergienko R, Landau D, Sheiner E. Fertility treatments and pediatric neoplasms of the offspring: results of a population-based cohort with a median follow-up of 10 years. Am J Obstet Gynecol. 2017 Mar;216(3):314.e1-314.e14. doi: 10.1016/j.ajog.2017.01.015. Epub 2017 Jan 30. | Duplication. |
| Mandy Spaan 2019 | 1. Spaan M, van den Belt-Dusebout AW, van den Heuvel-Eibrink MM, Hauptmann M, Lambalk CB, Burger CW, van Leeuwen FE; OMEGA-steering group. Risk of cancer in children and young adults conceived by assisted reproductive technology. Hum Reprod. 2019 Apr 1;34(4):740-750. doi: 10.1093/humrep/dey394. | Duplication. |
| Maayan Hagbi Bal 2021 | 1. Bal MH, Harlev A, Sergienko R, Levitas E, Har-Vardi I, Zeadna A, Mark-Reich A, Becker H, Ben-David N, Naggan L, Wainstock T. Possible association between in vitro fertilization technologies and offspring neoplasm. Fertil Steril. 2021 Jul;116(1):105-113. doi: 10.1016/j.fertnstert.2020.12.013. Epub 2021 Feb 14. | Duplication. |
| Tal Shabtai 2023 | 1. Shabtai T, Sheiner E, Wainstock T, Raziel A, Kessous R. Infertility Treatments Resulting in Twin Pregnancy: Does It Increase the Risk for Future Childhood Malignancy. J Clin Med. 2023 May 29;12(11):3728. doi: 10.3390/jcm12113728. | Duplication. |
| Susan E Puumala 2012 | 1. Puumala SE, Ross JA, Feusner JH, Tomlinson GE, Malogolowkin MH, Krailo MD, Spector LG. Parental infertility, infertility treatment and hepatoblastoma: a report from the Children's Oncology Group. Hum Reprod. 2012 Jun;27(6):1649-56. doi: 10.1093/humrep/des109. Epub 2012 Apr 3. | Wrong outcome. |
| P Rufat 1994 | 1. Rufat P, Olivennes F, de Mouzon J, Dehan M, Frydman R. Task force report on the outcome of pregnancies and children conceived by in vitro fertilization (France: 1987 to 1989). Fertil Steril. 1994 Feb;61(2):324-30. | Wrong exposure. |
| A M Michalek 1996 | 1. Michalek AM, Buck GM, Nasca PC, Freedman AN, Baptiste MS, Mahoney MC. Gravid health status, medication use, and risk of neuroblastoma. Am J Epidemiol. 1996 May 15;143(10):996-1001. doi: 10.1093/oxfordjournals.aje.a008682. | Wrong exposure. |
| E Roman 1997 | 1. Roman E, Ansell P, Bull D. Leukaemia and non-Hodgkin's lymphoma in children and young adults: are prenatal and neonatal factors important determinants of disease? Br J Cancer. 1997;76(3):406-15. doi: 10.1038/bjc.1997.399. | Wrong exposure. |
| A F Olshan 1999 | 1. Olshan AF, Smith J, Cook MN, Grufferman S, Pollock BH, Stram DO, Seeger RC, Look AT, Cohn SL, Castleberry RP, Bondy ML. Hormone and fertility drug use and the risk of neuroblastoma: a report from the Children's Cancer Group and the Pediatric Oncology Group. Am J Epidemiol. 1999 Nov 1;150(9):930-8. doi: 10.1093/oxfordjournals.aje.a010101. | Wrong exposure. |
| J Schüz 1999 | 1. Schüz J, Kaatsch P, Kaletsch U, Meinert R, Michaelis J. Association of childhood cancer with factors related to pregnancy and birth. Int J Epidemiol. 1999 Aug;28(4):631-9. doi: 10.1093/ije/28.4.631. | Wrong exposure. |
| Louise A Brinton 2004 | 1. Brinton LA, Krüger Kjaer S, Thomsen BL, Sharif HF, Graubard BI, Olsen JH, Bock JE. Childhood tumor risk after treatment with ovulation-stimulating drugs. Fertil Steril. 2004 Apr;81(4):1083-91. doi: 10.1016/j.fertnstert.2003.08.042. | Wrong exposure. |
| Ali Ayhan 2004 | 1. Ayhan A, Salman MC, Celik H, Dursun P, Ozyuncu O, Gultekin M. Association between fertility drugs and gynecologic cancers, breast cancer, and childhood cancers. Acta Obstet Gynecol Scand. 2004 Dec;83(12):1104-11. doi: 10.1111/j.0001-6349.2004.00669.x. | Wrong exposure. |
| Susan E Puumala 2007 | 1. Puumala SE, Ross JA, Olshan AF, Robison LL, Smith FO, Spector LG. Reproductive history, infertility treatment, and the risk of acute leukemia in children with down syndrome: a report from the Children's Oncology Group. Cancer. 2007 Nov 1;110(9):2067-74. doi: 10.1002/cncr.23025. | Wrong exposure. |
| Susan E Puumala 2010 | 1. Puumala SE, Spector LG, Wall MM, Robison LL, Heerema NA, Roesler MA, Ross JA. Infant leukemia and parental infertility or its treatment: a Children's Oncology Group report. Hum Reprod. 2010 Jun;25(6):1561-8. doi: 10.1093/humrep/deq090. Epub 2010 Apr 10. | Wrong exposure. |
| Susan E Puumala 2011 | 1. Puumala SE, Ross JA, Wall MM, Spector LG. Pediatric germ cell tumors and parental infertility and infertility treatment: a Children's Oncology Group report. Cancer Epidemiol. 2011 Oct;35(5):e25-31. doi: 10.1016/j.canep.2011.01.009. Epub 2011 Apr 6. | Wrong exposure. |
| Yi-Ru Tsai 2013 | 1. Tsai YR, Lan KC, Kung FT, Lin PY, Chiang HJ, Lin YJ, Huang FJ. The effect of advanced paternal age on the outcomes of assisted reproductive techniques among patients with azoospermia using cryopreserved testicular spermatozoa. Taiwan J Obstet Gynecol. 2013 Sep;52(3):351-5. doi: 10.1016/j.tjog.2013.06.001. | Wrong exposure. |
| Marie Hargreave 2015 | 1. Hargreave M, Jensen A, Nielsen TS, Colov EP, Andersen KK, Pinborg A, Kjaer SK. Maternal use of fertility drugs and risk of cancer in children--a nationwide population-based cohort study in Denmark. Int J Cancer. 2015 Apr 15;136(8):1931-9. doi: 10.1002/ijc.29235. Epub 2014 Oct 6. | Wrong exposure. |
| Barbara Luke 2016 | 1. Luke B, Brown MB, Missmer SA, Spector LG, Leach RE, Williams M, Koch L, Smith YR, Stern JE, Ball GD, Schymura MJ. Assisted reproductive technology use and outcomes among women with a history of cancer. Hum Reprod. 2016 Jan;31(1):183-9. doi: 10.1093/humrep/dev288. Epub 2015 Nov 17. | Wrong exposure. |
| M J Davies 2016 | 1. Davies MJ, Rumbold AR, Whitrow MJ, Willson KJ, Scheil WK, Mol BW, Moore VM. Spontaneous loss of a co-twin and the risk of birth defects after assisted conception. J Dev Orig Health Dis. 2016 Dec;7(6):678-684. doi: 10.1017/S2040174416000301. Epub 2016 Jul 4. | Wrong exposure. |
| Paolo Emanuele Levi-Setti 2018 | 1. Levi-Setti PE, Negri L, Baggiani A, Morenghi E, Albani E, Parini V, Cafaro L, Dioguardi CMC, Cesana A, Smeraldi A, Santoro A. Delayed childbearing and female ageing impair assisted reproductive technology outcome in survivors of male haematological cancers. J Assist Reprod Genet. 2018 Nov;35(11):2049-2056. doi: 10.1007/s10815-018-1283-5. Epub 2018 Aug 10. | Wrong exposure. |
| B Valenzuela-Alcaraz 2019 | 1. Valenzuela-Alcaraz B, Serafini A, Sepulveda-Martínez A, Casals G, Rodríguez-López M, Garcia-Otero L, Cruz-Lemini M, Bijnens B, Sitges M, Balasch J, Gratacós E, Crispi F. Postnatal persistence of fetal cardiovascular remodelling associated with assisted reproductive technologies: a cohort study. BJOG. 2019 Jan;126(2):291-298. doi: 10.1111/1471-0528.15246. Epub 2018 Jun 13. | Wrong exposure. |
| Greta Sommerhäuser 2021 | 1. Sommerhäuser G, Borgmann-Staudt A, Astrahantseff K, Baust K, Calaminus G, Dittrich R, Fernández-González MJ, Hölling H, König CJ, Schilling R, Schuster T, Lotz L, Balcerek M. Health outcomes in offspring born to survivors of childhood cancers following assisted reproductive technologies. J Cancer Surviv. 2021 Apr;15(2):259-272. doi: 10.1007/s11764-020-00929-0. Epub 2020 Aug 26. | Wrong exposure. |
| Anja Borgmann-Staudt 2022 | 1. Borgmann-Staudt A, Michael S, Sommerhaeuser G, Fernández-González MJ, Friedrich LA, Klco-Brosius S, Kepak T, Kruseova J, Michel G, Panasiuk A, Schmidt S, Lotz L, Balcerek M. The Use of Assisted Reproductive Technology by European Childhood Cancer Survivors. Curr Oncol. 2022 Aug 15;29(8):5748-5762. doi: 10.3390/curroncol29080453. | Wrong exposure. |
| Toshiaki Yasuoka 2022 | 1. Yasuoka T, Iwama N, Ota K, Hasegawa J, Metoki H, Saito M, Sugiyama T, Suzuki N. Pregnancy outcomes among female childhood, adolescent, and young adult cancer survivors assessed using internet-based nationwide questionnaire surveys in Japan. J Matern Fetal Neonatal Med. 2022 Dec;35(26):10667-10675. doi: 10.1080/14767058.2022.2155037IF: 1.7 Q3 . Epub 2022 Dec 25. | Wrong exposure. |
| Kimberly W Keefe 2024 | 1. Keefe KW, Lanes A, Stratton K, Green DM, Chow EJ, Oeffinger KC, Barton S, Diller L, Yasui Y, Leisenring WM, Armstrong GT, Ginsburg ES. Assisted reproductive technology use and outcomes in childhood cancer survivors. Cancer. 2024 Jan 1;130(1):128-139. doi: 10.1002/cncr.34995. Epub 2023 Sep 21. | Wrong exposure. |
| Roya Farhadi 2013 | 1. Farhadi R, Kazemi SH. Harlequin ichthyosis in a neonate born with assisted reproductive technology: a case report. Med J Islam Repub Iran. 2013 Nov;27(4):229-32. | Case report. |
| Antonia Tocino 2015 | 1. Tocino A, Blasco V, Prados N, Vargas MJ, Requena A, Pellicer A, Fernández-Sánchez M. Monozygotic twinning after assisted reproductive technologies: a case report of asymmetric development and incidence during 19 years in an international group of in vitro fertilization clinics. Fertil Steril. 2015 May;103(5):1185-9. doi: 10.1016/j.fertnstert.2015.01.033. Epub 2015 Feb 20. | Case report. |
| Ji Yoon Han 2016 | 1. Han JY, Park J, Jang W, Chae H, Kim M, Kim Y. A twin sibling with Prader-Willi syndrome caused by type 2 microdeletion following assisted reproductive technology: A case report. Biomed Rep. 2016 Jul;5(1):18-22. doi: 10.3892/br.2016.675. Epub 2016 May 12. | Case report. |
| V Arun Muthuvel 2018 | 1. Muthuvel VA, Ravindran M, Chander VA, Gounder CV. Live birth following in vitro fertilization in a breast cancer survivor: A case report and review of literature. J Cancer Res Ther. 2018 Jul-Sep;14(5):1157-1159. doi: 10.4103/0973-1482.174556. | Case report. |
| Jessica E M Dunleavy 2022 | 1. Dunleavy JEM, Dinh DT, Filby CE, Green E, Hofstee P, Pini T, Rivers N, Skerrett-Byrne DA, Wijayarathna R, Winstanley YE, Zhou W, Richani D. Reproductive biology research down under: highlights from the Australian and New Zealand Annual Meeting of the Society for Reproductive Biology, 2021. Reprod Fertil Dev. 2022 Aug;34(13):855-866. doi: 10.1071/RD22115. | Conference summary. |
| Laura A Schieve 2004 | 1. Schieve LA, Rasmussen SA, Buck GM, Schendel DE, Reynolds MA, Wright VC. Are children born after assisted reproductive technology at increased risk for adverse health outcomes? Obstet Gynecol. 2004 Jun;103(6):1154-63. doi: 10.1097/01.AOG.0000124571.04890.67. | Review. |
| Eamonn R Maher 2005 | 1. Maher ER. Imprinting and assisted reproductive technology. Hum Mol Genet. 2005 Apr 15;14 Spec No 1:R133-8. doi: 10.1093/hmg/ddi107. | Review. |
| E Basatemur 2008 | 1. Basatemur E, Sutcliffe A. Follow-up of children born after ART. Placenta. 2008 Oct;29 Suppl B:135-40. doi: 10.1016/j.placenta.2008.08.013. | Review. |
| Mukhopadhaya Neelanjana 2008 | 1. Neelanjana M, Sabaratnam A. Malignant conditions in children born after assisted reproductive technology. Obstet Gynecol Surv. 2008 Oct;63(10):669-76. doi: 10.1097/OGX.0b013e318181a9f0. | Review. |
| Tim Savage 2011 | 1. Savage T, Peek J, Hofman PL, Cutfield WS. Childhood outcomes of assisted reproductive technology. Hum Reprod. 2011 Sep;26(9):2392-400. doi: 10.1093/humrep/der212. | Review. |
| Susan Thrane 2013 | 1. Thrane S. Effectiveness of integrative modalities for pain and anxiety in children and adolescents with cancer: a systematic review. J Pediatr Oncol Nurs. 2013 Nov-Dec;30(6):320-32. doi: 10.1177/1043454213511538. | Review. |
| Yue-hong Lu 2013 | 1. Lu YH, Wang N, Jin F. Long-term follow-up of children conceived through assisted reproductive technology. J Zhejiang Univ Sci B. 2013 May;14(5):359-71. doi: 10.1631/jzus.B1200348. | Review. |
| E S Jungheim 2013 | 1. Jungheim ES, Schon SB, Schulte MB, DeUgarte DA, Fowler SA, Tuuli MG. IVF outcomes in obese donor oocyte recipients: a systematic review and meta-analysis. Hum Reprod. 2013 Oct;28(10):2720-7. doi: 10.1093/humrep/det292. Epub 2013 Jul 11. | Review. |
| Nanette Okun 2014 | 1. Okun N, Sierra S; GENETICS COMMITTEE; SPECIAL CONTRIBUTORS. Pregnancy outcomes after assisted human reproduction. J Obstet Gynaecol Can. 2014 Jan;36(1):64-83. doi: 10.1016/S1701-2163(15)30685-X. | Review. |
| Simon McDowell 2014 | 1. McDowell S, Kroon B, Ford E, Hook Y, Glujovsky D, Yazdani A. Advanced sperm selection techniques for assisted reproduction. Cochrane Database Syst Rev. 2014 Oct 28;(10):CD010461. doi: 10.1002/14651858.CD010461. | Review. |
| Federica Tomao 2014 | 1. Tomao F, Lo Russo G, Spinelli GP, Tomao S. Clinical use of fertility agents and risk of breast cancer: a recent update for an old problem. Curr Opin Obstet Gynecol. 2014 Jun;26(3):130-7. doi: 10.1097/GCO.0000000000000067. | Review. |
| S E Gellert 2018 | 1. Gellert SE, Pors SE, Kristensen SG, Bay-Bjørn AM, Ernst E, Yding Andersen C. Transplantation of frozen-thawed ovarian tissue: an update on worldwide activity published in peer-reviewed papers and on the Danish cohort. J Assist Reprod Genet. 2018 Apr;35(4):561-570. doi: 10.1007/s10815-018-1144-2. Epub 2018 Mar 1. | Review. |
| Alessandro Crocoli 2022 | 1. Crocoli A, Martucci C, Persano G, De Pasquale MD, Serra A, Accinni A, Aloi IP, Bertocchini A, Frediani S, Madafferi S, Pardi V, Inserra A. Vascular Access in Pediatric Oncology and Hematology: State of the Art. Children (Basel). 2022 Jan 5;9(1):70. doi: 10.3390/children9010070. | Review. |
| Germán Velez-Florez 2018 | 1. Velez-Florez G, Velez-Florez MC, Mantilla-Rivas JO, Patarroyo-Rodríguez L, Borrero-León R, Rodríguez-León S. Mind-Body Therapies in Childhood Cancer. Curr Psychiatry Rep. 2018 Jul 21;20(8):58. doi: 10.1007/s11920-018-0927-6. | Review. |

# eTable 2. Meta-analyses of risk estimates for cancers among children conceived via Assisted reproductive technology.

| **Outcomes** | **No. of studies** | **RR (95% CI)** | **p-value** | **Test for heterogeneity** | | **Test of publication bias** | |
| --- | --- | --- | --- | --- | --- | --- | --- |
|  |  |  |  | **I^2^ (%)** | **p-value** | **t** | **p-value** |
| Overall cancer | 15 | 1.21 (1.11–1.33) | ＜0.001 | 56.08 | 0.004 | 1.18 | 0.258 |
| Haematological malignancies | 14 | 1.16 (1.05–1.28) | ＜0.001 | 13.93 | 0.30 | 2.16 | 0.052 |
| Neural tumors | 12 | 1.19 (1.07–1.32) | ＜0.001 | 0.00 | 0.61 | -0.14 | 0.891 |
| Other solid tumors | 13 | 1.48 (1.26–1.73) | ＜0.001 | 64.83 | ＜0.001 | 1.54 | 0.151 |
| Leukemia | 12 | 1.16 (1.03–1.32) | 0.02 | 29.19 | 0.16 | 1.10 | 0.299 |
| Lymphoma | 8 | 1.12 (0.90–1.39) | 0.31 | 0.00 | 0.93 | 1.21 | 0.267 |
| ALL | 3 | 1.14 (0.95–1.36) | 0.16 | 0.00 | 0.43 | -0.31 | 0.810 |
| AML | 4 | 1.25 (0.72–2.18) | 0.42 | 32.32 | 0.22 | 0.42 | 0.717 |
| CNS tumors | 12 | 1.22 (1.09–1.38) | ＜0.001 | 0.00 | 0.58 | -1.17 | 0.271 |
| Peripheral Nervous Cell tumors | 8 | 1.01 (0.79–1.29) | 0.93 | 0.00 | 0.72 | 0.39 | 0.712 |
| Retinoblastoma | 10 | 1.74 (1.15–2.63) | 0.01 | 52.86 | 0.02 | 1.23 | 0.252 |
| Hepatic tumors | 5 | 2.73 (1.90–3.91) | ＜0.001 | 0.00 | 0.82 | -0.63 | 0.574 |
| Renal tumors | 10 | 1.27 (0.98–1.65) | 0.07 | 6.14 | 0.38 | 0.98 | 0.354 |
| Bone tumors and extraosseous sarcomas | 7 | 1.62 (1.26–2.07) | ＜0.001 | 6.15 | 0.38 | -1.46 | 0.194 |
| Germ cell tumors | 2 | 0.60 (0.25–1.42) | 0.24 | 0.00 | 0.97 | -0.14 | 0.913 |
| Embryonal tumors | 3 | 1.14 (0.99–1.32) | 0.06 | 0.00 | 0.43 | 0.40 | 0.759 |
| Epithelial tumors and melanoma | 4 | 1.67 (1.22–2.29) | ＜0.001 | 0.00 | 0.94 | -0.77 | 0.499 |

Abbreviations: ALL, acute lymphoblastic leukemia; AML, acute myelocytic leukemia; CI, confidence interval; CNS, central nervous system; RR, Relative risk.

p < 0.001: instead of statistical analysis results, p=0.000.

In this study, we standardized integration based on methodological specifications for multiple tumor outcomes across the 16 included studies. Referring to the classification framework proposed by Wang et al. [1] and adhering strictly to the International Classification of Childhood Cancer (Third Edition) (ICCC-3) criteria [2], we systematically grouped specific tumor types at a higher level of histopathological classification. Specifically, tumors of hematopoietic origin, such as acute lymphoblastic leukemia and acute myeloid leukemia, were grouped with lymphomas under 'hematological malignancies'; neuroblastomas were classified under 'neural tumors'; and the remainder of the non-hematological and non-neurological solid tumors was collectively categorized as 'other solid tumors'. This classification strategy aims to enhance statistical power and more accurately reveal potential associations between ART and childhood cancer risk by increasing the aggregation of similar tumor samples.

[1] Wang, T., Chen, L., Yang, T., Wang, L., Zhao, L., Zhang, S., Ye, Z., Chen, L., Zheng, Z., & Qin, J. (2019). Cancer risk among children conceived by fertility treatment. International journal of cancer, 144(12), 3001–3013. <https://doi.org/10.1002/ijc.32062>.

[2] Steliarova-Foucher, E., Stiller, C., Lacour, B., & Kaatsch, P. (2005). International Classification of Childhood Cancer, third edition. Cancer, 103(7), 1457–1467. <https://doi.org/10.1002/cncr.20910>.

# eTable 3. NEWCASTLE - OTTAWA QUALITY ASSESSMENT SCALE (COHORT STUDIES)

| Author, year | Selection | | | | Comparability | Outcome | | | Overall  quality |
| --- | --- | --- | --- | --- | --- | --- | --- | --- | --- |
|  | Representative of cohort | Selection of cohort | Exposure ascertainment | No history of disease | Comparability of cohorts | Outcome assessment | Follow-up long enough(median ≥ 4 years) | Adequacy of follow up |  |
| Paula Rios 2024 | 1 | 1 | 1 | 1 | 1 | 1 | 1 | 1 | 8 |
| Mandy Spaan 2023 | 1 | 1 | 1 | 1 | 1 | 1 | 1 | 1 | 8 |
| Shu Qin Wei 2022 | 1 | 1 | 1 | 1 | 1 | 1 | 1 | 1 | 8 |
| Shiue-Shan Weng 2022 | 1 | 1 | 1 | 1 | 1 | 1 | 1 | 1 | 8 |
| Nona Sargisian 2022 | 1 | 1 | 1 | 1 | 2 | 1 | 1 | 1 | 9 |
| Barbara Luke 2022 | 1 | 1 | 1 | 1 | 2 | 1 | 1 | 1 | 9 |
| Marie Hargreave 2019 | 1 | 1 | 1 | 1 | 1 | 1 | 1 | 1 | 8 |
| Logan G Spector 2019 | 1 | 1 | 1 | 1 | 1 | 1 | 1 | 1 | 8 |
| Carrie L Williams 2018 | 1 | 1 | 1 | 1 | 1 | 1 | 1 | 1 | 8 |
| Liat Lerner-Geva 2017 | 1 | 1 | 1 | 1 | 1 | 1 | 1 | 1 | 8 |
| Tamar Wainstock 2017 | 0 | 1 | 1 | 1 | 2 | 1 | 1 | 1 | 8 |
| Marte Myhre Reigstad 2016 | 1 | 1 | 1 | 1 | 2 | 1 | 1 | 1 | 9 |
| Carrie L Williams 2013 | 1 | 1 | 1 | 1 | 1 | 1 | 1 | 1 | 8 |
| Bengt Källén 2010 | 1 | 1 | 1 | 1 | 2 | 1 | 1 | 1 | 9 |
| Ojvind Lidegaard 2005 | 1 | 1 | 1 | 1 | 0 | 1 | 1 | 1 | 7 |
| F Bruinsma 2000 | 1 | 1 | 1 | 1 | 1 | 1 | 1 | 1 | 8 |

Wells GA, Shea D, O'Connell D, et al. The newcastle-ottawa scale (NOS) for assessing the quality of nonrandomised studies in meta-analyses. <https://www.ohri.ca/programs/clinical_epidemiology/oxford.asp>

**Selection**

1: Representativeness of the exposed cohort (1 point); 2: Selection of the non exposed cohort (1 point); 3: Ascertainment of exposure (1 point); 4: Demonstration that outcome of interest was not present at start of study (1 point).

**Comparability**

1: Comparability of cohorts on the basis of the design or analysis (2 points).

**Outcome**

1: Assessment of outcome (1 point); 2: Was follow-up long enough for outcomes to occur (1 point); 3: Adequacy of follow up of cohorts (1 point).

# eTable 4. Subgroup analysis for risk of overall cancer, haematological malignancies, neural tumors, other solid tumors, Leukemia, CNS tumors, Retinoblastoma and Renal tumors among children conceived by ART.

| **Subgroup variables** | **Overall cancer**  1.21 (1.11–1.33), p＜0.001;  I^2^ = 56.08%, p = 0.004 | **Haematological malignancies**  1.16 (1.05–1.28), p = 0.01;  I^2^ = 13.93%, p = 0.26 | **Neural tumors**  1.19 (1.07–1.32), p＜0.001;  I^2^ = 0.00%, p = 0.61 | **Other solid tumors**  1.48 (1.26–1.73), p < 0.001;  I^2^ = 64.83%, p < 0.001 |
| --- | --- | --- | --- | --- |
| **Geographic region** | TSD: χ^2^ = 3.46, p = 0.06 | TSD: χ^2^ = 0.34, p = 0.56 | TSD: χ^2^ = 1.35, p = 0.25 | TSD: χ^2^ = 2.53, p = 0.11 |
| Europe | 1.12 (1.05–1.21) (n = 8), p = 0.001;  I^2^ = 11.83%, p = 0.34 | 1.15 (1.03–1.27) (n = 9), p = 0.011;  I^2^ = 0.00%, p = 0.47 | 1.13 (1.00–1.29) (n = 8), p = 0.059;  I^2^ = 0.00%, p = 0.69. | 1.33 (1.12–1.57) (n = 9), p = 0.001;  I^2^ = 35.54%, p = 0.14 |
| Non–Europe | 1.34 (1.13–1.59) (n = 7), p = 0.001;  I^2^ = 61.61%, p = 0.01 | 1.24 (0.97–1.58) (n = 5), p = 0.082;  I^2^ = 41.36%, p = 0.13 | 1.28 (1.09–1.51) (n = 4), p = 0.003;  I^2^ = 0.00%, p = 0.42 | 1.76 (1.30–2.38) (n = 4), p < 0.001;  I^2^ = 81.39%, p < 0.001 |
| **Maternal age at conceiving** | TSD: χ^2^ = 1.91, p = 0.17 | TSD: χ^2^ = 0.00, p = 0.95 | TSD: χ^2^ = 0.84, p = 0.36 | TSD: χ^2^ = 0.96, p = 0.33 |
| ≥30 | 1.15 (1.03–1.28) (n = 8), p = 0.01;  I^2^ = 50.30%, p = 0.05 | 1.16 (0.99–1.36) (n = 8), p = 0.058;  I^2^ = 34.36%, p = 0.15 | 1.15 (1.01–1.31) (n = 7), p = 0.044;  I^2^ = 0.00%, p = 0.67 | 1.40 (1.17–1.67) (n = 7), p < 0.001;  I^2^ = 58.51%, p = 0.02 |
| ＜30 | 1.33 (1.12–1.57) (n = 6), p = 0.001;  I^2^ = 69.40%, p = 0.01 | 1.15 (1.01–1.32) (n = 5), p = 0.043;  I^2^ = 0.00%, p = 0.41 | 1.27 (1.07–1.52) (n = 5), p = 0.008;  I^2^ = 7.65%, p = 0.36 | 1.73 (1.17–2.56) (n = 5), p = 0.006;  I^2^ =78.83%, p < 0.001 |
| **Unexposed population** | TSD: χ^2^ = 1.31, p = 0.25 | TSD: χ^2^ = 0.33, p = 0.56 | TSD: χ^2^ = 1.36, p = 0.24 | TSD: χ^2^ = 0.07, p = 0.79 |
| Children conceived naturally | 1.27 (1.13–1.44) (n = 7), p ＜0.001;  I^2^ = 68.07%, p < 0.001 | 1.12 (0.99–1.27) (n = 7), p = 0.061;  I^2^ = 16.00%, p = 0.31 | 1.24 (1.10–1.39) (n = 6), p ＜0.001;  I^2^ = 0.00%, p = 0.60 | 1.50 (1.21–1.87) (n = 7), p < 0.001;  I^2^ =77.43%, p < 0.001 |
| The general population and children not conceived via ART | 1.13 (0.94–1.34) (n = 7), p = 0.188;  I^2^ = 49.41%, p = 0.08 | 1.22 (0.96–1.54) (n = 6), p < 0.109;  I^2^ = 24.29%, p = 0.26 | 1.06 (0.84–1.34) (n = 5), p = 0.634;  I^2^ = 2.04%, p = 0.38 | 1.57 (1.26–1.94) (n = 5), p < 0.001;  I^2^ = 0.00%, p = 0.41 |

| **Subgroup variables** | **Leukemia**  1.16 (1.03–1.32), p = 0.02;  I^2^ = 29.19%, p = 0.16 | **CNS tumors**  1.22 (1.09–1.38), p < 0.001;  I^2^ = 0.00%, p = 0.58 | **Retinoblastoma**  1.74 (1.15–2.63), p = 0.01;  I^2^ = 52.86%, p = 0.02 | **Renal tumors**  1.27 (0.98–1.65), p = 0.07;  I^2^ = 6.14%, p = 0.38 |
| --- | --- | --- | --- | --- |
| **Geographic region** | TSD: χ^2^ = 0.21, p = 0.65 | TSD: χ^2^ = 0.42, p = 0.52 | TSD: χ^2^ = 2.94, p = 0.09 | TSD: χ^2^ = 2.08, p = 0.15 |
| Europe | 1.15 (1.02–1.29) (n = 7), p = 0.023;  I^2^ =2.79%, p = 0.40 | 1.18 (1.02–1.36) (n = 8), p = 0.021;  I^2^ = 0.00%, p = 0.72 | 1.22 (0.90–1.66) (n = 7), p = 0.201;  I^2^ = 0.00%, p = 0.60 | 1.08 (0.79–1.49) (n = 7), p = 0.615;  I^2^ = 0.00%, p = 0.99 |
| Non–Europe | 1.23 (0.92–1.64) (n = 5), p = 0.155;  I^2^ = 51.87%, p = 0.06 | 1.30 (1.00–1.70) (n = 4), p = 0.052;  I^2^ = 16.42%, p = 0.31 | 2.54 (1.17–5.52) (n = 3), p = 0.019;  I^2^ = 70.83%, p = 0.02 | 1.91 (0.95–3.85) (n = 3), p = 0.070;  I^2^ = 56.71%, p = 0.07 |
| **Maternal age at conceiving** | TSD: χ^2^ = 0.04, p = 0.84 | TSD: χ^2^ = 0.58, p = 0.45 | TSD: χ^2^ = 0.72, p = 0.40 | TSD: χ^2^ = 0.83, p = 0.36 |
| ≥30 | 1.18 (0.99–1.41) (n = 8), p = 0.061;  I^2^ = 41.12%, p = 0.10 | 1.16 (0.99–1.36) (n = 7), p = 0.075;  I^2^ = 0.00%, p = 0.84 | 1.37 (0.94–1.99) (n = 5), p = 0.102;  I^2^ = 28.44%, p = 0.23 | 1.16 (0.84–1.58) (n = 6), p = 0.366;  I^2^ = 0.00%, p = 0.92 |
| ＜30 | 1.15 (0.95–1.39) (n = 4), p = 0.144;  I^2^ = 17.42%, p = 0.30 | 1.30 (1.01–1.67) (n = 5), p = 0.041;  I^2^ = 29.69%, p = 0.22 | 2.22 (0.77–6.37) (n = 4), p = 0.138;  I^2^ = 66.49%, p = 0.03 | 1.81 (0.73–4.52) (n = 3), p = 0.202;  I^2^ = 72.12%, p = 0.03 |
| **Unexposed population** | TSD: χ^2^ = 0.09, p = 0.76 | TSD: χ^2^ = 1.44, p = 0.23 | TSD: χ^2^ = 1.32, p = 0.25 | TSD: χ^2^ = 0.57, p = 0.45 |
| Children conceived naturally | 1.13 (0.96–1.32) (n = 6), p = 0.129;  I^2^ = 38.27%, p = 0.15 | 1.28 (1.12–1.47) (n = 6), p < 0.001;  I^2^ = 0.00%, p = 0.52 | 1.94 (1.09–3.43) (n = 6), p < 0.023;  I^2^ = 70.54%, p = 0.005 | 1.40 (0.93–2.11) (n = 6), p < 0.104;  I^2^ = 40.90%, p = 0.13 |
| The general population and children not conceived via ART | 1.19 (0.89–1.58) (n = 5), p = 0.234;  I^2^ = 36.44%, p = 0.19 | 1.07 (0.83–1.39) (n = 5), p = 0.612;  I^2^ = 0.00%, p = 0.40 | 1.06 (0.45–2.49) (n = 4), p = 0.891;  I^2^ = 0.00%, p = 0.79 | 1.05 (0.57–1.96) (n = 3), p = 0.870;  I^2^ = 0.00%, p = 0.57 |

ART, assisted reproductive technology; CNS, central nervous system; TSD, test for subgroup differences.

# eTable 5. Grade Grading Details

| **Outcomes** | **Relative effect difference (95 % CI)** | **Absolute effect difference (95 % CI)** | **No of Participants (studies)** | **Quality of the evidence (GRADE)** |
| --- | --- | --- | --- | --- |
| **Cancer outcome** |  |  |  |  |
| Overall cancer | RR = 1.21, 95 % CI | – | 29,628,386 | ⨁◯◯◯ |
|  | (1.11–1.33) |  | (15) | Very low (b-) |
| Haematological malignancies | RR = 1.16, 95 % CI | – | 29,380,950 | ⨁⨁◯◯ |
|  | (1.05–1.28) |  | (14) | Low |
| Neural tumors | RR = 1.19, 95 % CI | – | 28,134,895 | ⨁⨁◯◯ |
|  | (1.07–1.32) |  | (12) | Low |
| Other solid tumors | RR = 1.48, 95 % CI | – | 28,583,296 | ⨁⨁◯◯ |
|  | (1.26–1.73) |  | (13) | Low |
| Leukemia | RR = 1.16, 95 % CI | – | 26,487,979 | ⨁⨁◯◯ |
|  | (1.03–1.32) |  | (12) | Low |
| Lymphoma | RR = 1.12, 95 % CI | – | 24,053,610 | ⨁⨁◯◯ |
|  | (0.90–1.39) |  | (8) | Low |
| ALL | RR = 1.14, 95 % CI | – | 12,496,693 | ⨁⨁◯◯ |
|  | (0.95–1.36) |  | (3) | Low |
| AML | RR = 1.25, 95 % CI | – | 12,945,094 | ⨁⨁◯◯ |
|  | (0.72–2.18) |  | (4) | Low |
| CNS tumors | RR = 1.22, 95 % CI | – | 28,134,895 | ⨁⨁◯◯ |
|  | (1.09–1.38) |  | (12) | Low |
| Peripheral Nervous Cell tumors | RR = 1.01, 95 % CI | – | 15,556,205 | ⨁⨁◯◯ |
|  | (0.79–1.29) |  | (8) | Low |
| Retinoblastoma | RR = 1.74, 95 % CI | – | 26,028,035 | ⨁⨁⨁◯ |
|  | (1.15–2.63) |  | (10) | Moderate (a+) |
| Hepatic tumors | RR = 2.73, 95 % CI | – | 6,443,705 | ⨁⨁⨁◯ |
|  | (1.90–3.91) |  | (5) | Moderate (a+) |
| Renal tumors | RR = 1.27, 95 % CI | – | 23,672,714 | ⨁⨁◯◯ |
|  | (0.98–1.65) |  | (10) | Low |
| Bone tumors and extraosseous sarcomas | RR = 1.62, 95 % CI | – | 23,122,927 | ⨁⨁⨁◯ |
|  | (1.26–2.07) |  | (7) | Moderate (a+) |
| Germ cell tumors | RR = 1.00, 95 % CI | – | 4,802,910 | ⨁◯◯◯ |
|  | (0.47–2.16) |  | (3) | Very low (e-) |
| Embryonal tumors | RR = 1.14, 95 % CI | – | 12,386,600 | ⨁⨁◯◯ |
|  | (0.99–1.32) |  | (3) | Low |
| Epithelial tumors and melanoma | RR = 1.67, 95 % CI | – | 18,914,971 | ⨁⨁◯◯ |
|  | (1.22–2.29) |  | (4) | Low |
| **Subgroup variables** |  |  |  |  |
| **Overall cancer** |  |  |  |  |
| **Geographic region** |  |  |  |  |
| Europe | RR = 1.12, 95 % CI | – | 21,698,628 | ⨁⨁◯◯ |
|  | (1.05–1.21) |  | (8) | Low |
| Non-Europe | RR = 1.34, 95 % CI | – | 7,481,357 | ⨁◯◯◯ |
|  | (1.13–1.59) |  | (7) | Very low (b-) |
| **Maternal gestational age** |  |  |  |  |
| ≥30 | RR = 1.15, 95 % CI | – | 15,175,703 | ⨁◯◯◯ |
|  | (1.03–1.28) |  | (8) | Very low (b-) |
| ＜30 | RR = 1.33, 95 % CI | – | 13,999,033 | ⨁◯◯◯ |
|  | (1.12–1.57) |  | (6) | Very low (b-) |
| **Unexposed population** |  |  |  |  |
| Children conceived naturally | RR = 1.27, 95 % CI | – | 14,780,548 | ⨁◯◯◯ |
|  | (1.13–1.44) |  | (7) | Very low (b-) |
| The general population and children not conceived via ART | RR = 1.13, 95 % CI  (0.94–1.34) | – | 5,941,728  (7) | ⨁◯◯◯  Very low (b-) |
| **Haematological malignancies** |  |  |  |  |
| **Geographic region** |  |  |  |  |
| Europe | RR = 1.15, 95 % CI | – | 22,147,029 | ⨁⨁◯◯ |
|  | (1.03–1.27) |  | (9) | Low |
| Non-Europe | RR = 1.24, 95 % CI | – | 7,233,921 | ⨁⨁◯◯ |
|  | (0.97–1.58) |  | (5) | Low |
| **Maternal gestational age** |  |  |  |  |
| ≥30 | RR = 1.16, 95 % CI | – | 15,175,703 | ⨁⨁◯◯ |
|  | (0.99–1.36) |  | (8) | Low |
| ＜30 | RR = 1.15, 95 % CI | – | 13,756,846 | ⨁⨁◯◯ |
|  | (1.01–1.32) |  | (5) | Low |
| **Unexposed population** |  |  |  |  |
| Children conceived naturally | RR = 1.12, 95 % CI | – | 14,986,762 | ⨁⨁◯◯ |
|  | (0.99–1.27) |  | (7) | Low |
| The general population and children not conceived via ART | RR = 1.22, 95 % CI  (0.96–1.54) | – | 5,936,479  (6) | ⨁⨁◯◯  Low |
| **Neural tumors** |  |  |  |  |
| **Geographic region** |  |  |  |  |
| Europe | RR = 1.13, 95 % CI | – | 21,698,628 | ⨁⨁◯◯ |
|  | (1.00–1.29) |  | (8) | Low |
| Non-Europe | RR = 1.28, 95 % CI | – | 6,436,267 | ⨁⨁◯◯ |
|  | (1.09–1.51) |  | (4) | Low |
| **Maternal gestational age** |  |  |  |  |
| ≥30 | RR = 1.15, 95 % CI | – | 14,378,049 | ⨁⨁◯◯ |
|  | (1.01–1.31) |  | (7) | Low |
| ＜30 | RR = 1.27, 95 % CI | – | 13,756,846 | ⨁⨁◯◯ |
|  | (1.07–1.52) |  | (5) | Low |
| **Unexposed population** |  |  |  |  |
| Children conceived naturally | RR = 1.24, 95 % CI | – | 14,538,361 | ⨁⨁◯◯ |
|  | (1.10–1.39) |  | (6) | Low |
| The general population and children not conceived via ART | RR = 1.06, 95 % CI  (0.84–1.34) | – | 5,138,825  (5) | ⨁◯◯◯  Very low (d-) |
| **Other solid tumors** |  |  |  |  |
| **Geographic region** |  |  |  |  |
| Europe | RR = 1.33, 95 % CI | – | 22,147,029 | ⨁⨁◯◯ |
|  | (1.12–1.57) |  | (9) | Low |
| Non-Europe | RR = 1.76, 95 % CI | – | 6,436,267 | ⨁⨁◯◯ |
|  | (1.30–2.38) |  | (4) | Low |
| **Maternal gestational age** |  |  |  |  |
| ≥30 | RR = 1.40, 95 % CI | – | 14,378,049 | ⨁◯◯◯ |
|  | (1.17–1.67) |  | (7) | Very low (b-) |
| ＜30 | RR = 1.73, 95 % CI | – | 13,756,846 | ⨁◯◯◯ |
|  | (1.17–2.56) |  | (5) | Very low (b-) |
| **Unexposed population** |  |  |  |  |
| Children conceived naturally | RR = 1.50, 95 % CI | – | 14,986,762 | ⨁◯◯◯ |
|  | (1.21–1.87) |  | (7) | Very low (b-) |
| The general population and children not conceived via ART | RR = 1.57, 95 % CI  (1.26–1.94) | – | 5,138,825  (5) | ⨁⨁◯◯  Low |
| **Leukemia** |  |  |  |  |
| **Geographic region** |  |  |  |  |
| Europe | RR = 1.15, 95 % CI | – | 19,254,058 | ⨁⨁◯◯ |
|  | (1.02–1.29) |  | (7) | Low |
| Non-Europe | RR = 1.23, 95 % CI | – | 7,233,921 | ⨁◯◯◯ |
|  | (0.92–1.64) |  | (5) | Very low (b-) |
| **Maternal gestational age** |  |  |  |  |
| ≥30 | RR = 1.18, 95 % CI | – | 15,175,703 | ⨁⨁◯◯ |
|  | (0.99–1.41) |  | (8) | Low |
| ＜30 | RR = 1.15, 95 % CI | – | 11,312,276 | ⨁⨁◯◯ |
|  | (0.95–1.39) |  | (4) | Low |
| **Unexposed population** |  |  |  |  |
| Children conceived naturally | RR = 1.13, 95 % CI | – | 14,538,361 | ⨁⨁◯◯ |
|  | (0.96–1.32) |  | (6) | Low |
| The general population and children not conceived via ART | RR = 1.19, 95 % CI  (0.89–1.58) | – | 3,491,909  (5) | ⨁⨁◯◯  Low |
| **CNS tumors** |  |  |  |  |
| **Geographic region** |  |  |  |  |
| Europe | RR = 1.18, 95 % CI | – | 21,698,628 | ⨁⨁◯◯ |
|  | (1.02–1.36) |  | (8) | Low |
| Non-Europe | RR = 1.30, 95 % CI | – | 6,436,267 | ⨁⨁◯◯ |
|  | (1.00–1.70) |  | (4) | Low |
| **Maternal gestational age** |  |  |  |  |
| ≥30 | RR = 1.16, 95 % CI | – | 14,378,049 | ⨁⨁◯◯ |
|  | (0.99–1.36) |  | (7) | Low |
| ＜30 | RR = 1.30, 95 % CI | – | 13,756,846 | ⨁⨁◯◯ |
|  | (1.01–1.67) |  | (5) | Low |
| **Unexposed population** |  |  |  |  |
| Children conceived naturally | RR = 1.28, 95 % CI | – | 22,393,360 | ⨁⨁◯◯ |
|  | (1.12–1.47) |  | (6) | Low |
| The general population and children not conceived via ART | RR = 1.07, 95 % CI  (0.83–1.39) | – | 5,138,825  (5) | ⨁⨁◯◯  Low |
| **Retinoblastoma** |  |  |  |  |
| **Geographic region** |  |  |  |  |
| Europe | RR = 1.22, 95 % CI | – | 21,110,333 | ⨁⨁◯◯ |
|  | (0.90–1.66) |  | (7) | Low |
| Non-Europe | RR = 2.54, 95 % CI | – | 4,917,702 | ⨁◯◯◯ |
|  | (1.17–5.52) |  | (3) | Very low (b-) |
| **Maternal gestational age** |  |  |  |  |
| ≥30 | RR = 1.37, 95 % CI | – | 13,341,353 | ⨁⨁⨁◯ |
|  | (0.94–1.99) |  | (5) | Moderate (a+) |
| ＜30 | RR = 2.22, 95 % CI | – | 12,238,281 | ⨁◯◯◯ |
|  | (0.77–6.37) |  | (4) | Very low (b-) |
| **Unexposed population** |  |  |  |  |
| Children conceived naturally | RR = 1.94, 95 % CI | – | 21,323,196 | ⨁◯◯◯ |
|  | (1.09–3.43) |  | (6) | Very low (b-) |
| The general population and children not conceived via ART | RR = 1.06, 95 % CI  (0.45–2.49) | – | 4,191,378  (4) | ⨁◯◯◯  Very low (d-) |
| **Renal tumors** |  |  |  |  |
| **Geographic region** |  |  |  |  |
| Europe | RR = 1.08, 95 % CI | – | 18,755,012 | ⨁⨁◯◯ |
|  | (0.79–1.49) |  | (7) | Low |
| Non-Europe | RR = 1.91, 95 % CI | – | 4,917,702 | ⨁◯◯◯ |
|  | (0.95–3.85) |  | (3) | Very low (d-) |
| **Maternal gestational age** |  |  |  |  |
| ≥30 | RR = 1.16, 95 % CI | – | 13,430,602 | ⨁⨁◯◯ |
|  | (0.84–1.58) |  | (6) | Low |
| ＜30 | RR = 1.81, 95 % CI | – | 9,793,711 | ⨁◯◯◯ |
|  | (0.73–4.52) |  | (3) | Very low (d-) |
| **Unexposed population** |  |  |  |  |
| Children conceived naturally | RR = 1.40, 95 % CI | – | 21,323,196 | Low |
|  | (0.93–2.11) |  | (6) | ⨁◯◯◯ |
| The general population and children not conceived via ART | RR = 1.05, 95 % CI  (0.57–1.96) | – | 1,746,808  (3) | Low  ⨁◯◯◯ |
| **ART type** |  |  |  |  |
| In vitro fertilization | RR = 1.13, 95 % CI | – | 6,065,182 | Low |
|  | (0.96–1.32) |  | (5) | ⨁◯◯◯ |

Note:

Abbreviations: ALL, acute lymphoblastic leukemia; AML, acute myelocytic leukemia; ART, assisted reproductive technology; CI, confidence interval; CNS, central nervous system; RR, relative risk.

**Cohort studies are usually evaluated from a low level (⨁⨁◯◯).**

⨁⨁⨁◯: Evidence of moderate quality suggests that the current efficacy evaluation results are likely to be close to the true value;

⨁⨁◯◯: Low quality research evidence suggests that the reliability of current efficacy evaluation results is uncertain;

⨁◯◯◯: Very low quality research evidence, suggesting uncertainty about the reliability of current efficacy evaluation results;

a-: Risk of bias in the study; b-: Inconsistencies between studies; c-: indirectness; d-: Accuracy of the findings; e-: Publication bias or small sample size

a+: Based on consistent evidence from two or more observational studies with no known confounders, RR > 2 (< 0.5) (+1); b+: Direct evidence based on real and no significant threat RR > 2 (< 0.5) (+2); c+: Evidence of dose-response gradient (+1); d+: All known confounders reduced the effect (+1).
